# Synergistic efficacy of simultaneous anti-TGF-β/VEGF bispecific antibody and PD-1 blockade in cancer therapy

**DOI:** 10.1186/s13045-023-01487-5

**Published:** 2023-08-12

**Authors:** Mengke Niu, Ming Yi, Yuze Wu, Lijuan Lyu, Qing He, Rui Yang, Liang Zeng, Jian Shi, Jing Zhang, Pengfei Zhou, Tingting Zhang, Qi Mei, Qian Chu, Kongming Wu

**Affiliations:** 1 Department of Oncology, Tongji Hospital of Tongji Medical College, Huazhong University of Science and Technology, Wuhan, 430030 China; 2 Department of Breast Surgery, The First Affiliated Hospital, College of Medicine, Zhejiang University, Hangzhou, 310000 China; 3 Department of Oncology, The Second Affiliated Hospital of Xi’an Jiaotong University, Xi’an, 710000 China; 4 Wuhan YZY Biopharma Co., Ltd, Biolake, C2-1, No.666 Gaoxin Road, Wuhan, 430075 People’s Republic of China; 5 Cancer Center, Shanxi Bethune Hospital, Shanxi Academy of Medical Science, Tongji Shanxi Hospital, Third Hospital of Shanxi Medical University, Taiyuan, 030032 China; 6 Cancer Center, Tongji Hospital of Tongji Medical College, Huazhong University of Science and Technology, Wuhan, 430030 China

**Keywords:** TGF-β, VEGF, Bispecific antibody, PD-1, Cancer immunotherapy, The tumor microenvironment

## Abstract

**Background:**

Recently, therapeutic antibodies against programmed cell death 1 (PD-1) and its ligand (PD-L1) have exerted potent anticancer effect in a variety of tumors. However, blocking the PD-1/PD-L1 axis alone is not sufficient to restore normal immune response. Other negative regulators of antitumor immunity, like TGF-β and VEGFA, are also involved in immune escape of tumor cells and induce immunotherapy resistance.

**Methods:**

We developed a novel anti-TGF-β/VEGF bispecific antibody Y332D based on the Nano-YBODY™ technology platform. The CCK-8, flow cytometry, SBE4 luciferase reporter assay, western blotting and transwell assays were used to measure the biological activities of the anti-TGF-β moiety. The NFAT luciferase reporter assay, luminescent cell viability assay and tube formation assay were used to measure the biological activities of the anti-VEGF moiety. The in vivo anticancer efficacy of Y332D alone or in combination with PD-1 blockade was evaluated in H22, EMT-6, 4T1, and AKT/Ras-driven murine hepatocellular carcinoma tumor models. Immunofluorescent staining, flow cytometry, RNA-seq and quantitative RT-PCR were adopted to analyze the alterations in the tumor microenvironment.

**Results:**

Y332D could maintain specific binding affinities for TGF-β and VEGFA. Y332D almost entirely counteracted the in vitro biological functions of TGF-β and VEGFA, including immunosuppression, activated TGF-β signaling, epithelial-mesenchymal transition (EMT), activated VEGF/VEGFR signaling, HUVEC proliferation and tube formation. The in vivo experiment data demonstrated that Y332D was more effective in inhibiting tumor growth and metastasis than anti-TGF-β and anti-VEGF monotherapies. In combination therapies, Y332D plus PD-1 blockade exhibited the most potent and durable anticancer effect. Mechanistically, Y332D plus PD-1 blockade upregulated the density and function of tumor-infiltrating lymphocytes and exerted reinvigorated antitumor immunity.

**Conclusion:**

Y332D could simultaneously block TGF-β and VEGF signalings. In comparison with the monotherapies, Y332D combined with PD-1 blockade exerts superior antitumor effect through improving immune microenvironment.

**Supplementary Information:**

The online version contains supplementary material available at 10.1186/s13045-023-01487-5.

## Background

Programmed cell death protein 1 (PD-1)/programmed death-ligand 1 (PD-L1) has been identified as a critical pathway involved in tumor immune escape [1–3]. The binding of PD-1 and its ligand PD-L1 inhibits the phosphorylation of T cell receptor (TCR) and CD28 downstream signaling molecules and leads to T cell exhaustion [4, 5]. Blockade of PD-1/PD-L1 can restore T cell activity and enhance the immune response [6]. Therefore, immunotherapy represented by PD-1/PD-L1 antibodies has made significant breakthroughs in anticancer therapeutics. However, only a small number of individuals can benefit from PD-1/PD-L1 antibodies, and blocking PD-1/PD-L1 signaling alone is insufficient to normalize the complex immune microenvironment [7, 8]. The antitumor immune response involves several steps in the cancer-immunity cycle, including the release of tumor antigens, antigen presentation, T cell priming and activation, transport and infiltration of T cells to the tumor site, and recognition and eradication of tumor cells by T cells [9]. Aside from PD-1/PD-L1, various other negative factors including TGF-β and VEGF also regulate antitumor immunity [9, 10].

The actively metabolic tumor cells and insufficient blood supply lead to the hypoxic tumor microenvironment, which in turn responds to elevated levels of certain pro-angiogenic factors like VEGFA [11, 12]. VEGF stimulates the proliferation of endothelial cells and promotes angiogenesis [13]. High VEGF concentrations, however, impede tumor vascular maturation [14]. Increased intertissue fluid pressure caused by immature and highly permeable arteries hinders the trafficking and infiltration of immune cells to the tumor site [15]. Additionally, VEGF has been reported to exert immunosuppressive properties [16, 17]. Upregulated VEGF promotes T cell depletion, inhibits the maturation of dendritic cell (DC), and promotes the recruitment of Treg, myeloid-derived suppressor cell (MDSC) and pro-tumor M2 tumor-associated macrophages (TAM) [18–20]. Immunosuppressive cells and secreted cytokines are critical factors mediating tumor progression and immune resistance [21]. However, the clinical benefit of anti-angiogenic therapy has still been limited [22, 23]. In numerous basic and clinical studies, the combination of anti-vascular targeting drugs with PD-1/PD-L1 antibodies has demonstrated notably synergistic antitumor effect in a variety type of cancer [24–28].

TGF-β is a multi-functional cytokine that is produced by tumor cells, immune cells and mesenchymal cells [29]. TGF-β is associated with a poor disease prognosis in advanced tumors because it promotes distant metastasis of tumor cells and resistance to therapy [30, 31]. High levels of TGF-β regulate the activities of multiple immune cells within the tumor microenvironment by impairing T and NK cell function, inhibiting antigen presentation by DCs and inducing Treg differentiation [32, 33]. TGF-β in the tumor microenvironment also regulates the activity of cancer-associated fibroblasts (CAF) and impedes lymphocyte infiltration by promoting the production of peritumor collagen [30, 34, 35]. TGF-β pathway antagonists are rapidly emerging as highly promising, safe and effective anticancer agents, and their safety and efficacy have been evaluated in trials [36]. Besides, higher expression of the *TGFB1* gene was observed in tumor tissues from patients who resist to PD-1/PD-L1 antibodies [37]. TGF-β blockade could improve the therapeutic efficacy of PD-1/PD-L1 antibodies and attenuate immunotherapeutic resistance [38, 39].

Even while the combination of PD-1/PD-L1 antibodies with anti-vascular or anti-TGF-β targeting drugs has greatly improved treatment efficiency in clinical studies, some patients remain unresponsive to therapy. It was reported that CAF elevated the expression of VEGF, TGF-β, and PD-L1 in a hypoxic tumor microenvironment [40]. This expression pattern suggests that VEGF, TGF-β and PD-L1 triple blockage may be more efficient in modulating the immune microenvironment than double blockade of VEGF combined with PD-1 or TGF-β combined with PD-1.

In order to optimize the antitumor efficacy of PD-1/PD-L1 antibodies, we developed the first novel anti-TGF-β/VEGF bispecific antibody Y332D, which can simultaneously block both immune-negative signals TGF-β and VEGF in the tumor microenvironment. The Nano-YBODY™ technology platform used for Y332D development was designed by Wuhan YZY Biopharma, featuring high production yield and structural stability. In this study, we explored the in vitro biochemistry characteristics of Y332D and evaluated the in vivo antitumor activities of Y332D alone or in combination with PD-1 blockade. We also analyzed the immune profile within the tumor microenvironment to identify the synergistic mechanism of combination therapy.

## Materials and methods

### Cell lines and therapeutic antibodies

Murine cancer cell lines 4T1 (breast cancer), EMT-6 (breast cancer), H22 (hepatocellular carcinoma) were cultured with RPMI-1640 (Gibco) containing 10% fetal bovine serum (FBS) (Excell). Human cancer cell line A549 (lung cancer) was cultured with F12-K (21127022, Gibco) containing 10% FBS. Human cancer cell line MDA-MB-231 (breast cancer) was cultured with DMEM (Gibco) containing 10% FBS. Murine T cell lines CTLL-2 and HT-2 were cultured in RPMI-1640 (ATCC modification, containing glutathione and vitamins) (A10491-01, Gibco) with 10% FBS and 200 IU/ml interleukin-2 (IL-2, Beijing Fourrings). 293-NFAT was cultured with MEM (12561-056, Gibco) containing 10% FBS. HUVEC (human umbilical vein endothelial cell) was cultured with ECM (1001, Sciencell) containing 10% FBS.

The therapeutic antibodies including anti-VEGF, anti-TGF-β, anti-PD-1, Y332D (anti-VEGF/TGF-β bispecific antibody) and isotype control antibody (human IgG) in this study were provided by Wuhan YZY Biopharma.

### Sodium dodecyl-sulfate polyacrylamide gel electrophoresis (SDS-PAGE)

The prepared Y332D was analyzed by SDS-PAGE with Coomassie Brilliant Blue staining. To verify the molecular weight of Y332D, non-reduced and reduced SDS-PAGE were performed. The non-reduced sample was prepared by mixing the protein with 5 μl of 0.5 M 2-Iodoacetamide (IAM) solution and incubating for 30 min away from light, then mixing the sample with 10 μl of NR Sample Buffer and incubating for 5 min at 60 °C. The reduced sample was prepared by mixing the protein with 10 μl of R Sample Buffer and incubating for 10 min at 70 °C. The sample was separated via 4–20% SurePAGE. After the SDS-PAGE gel was stained with Coomassie Brilliant Blue and decolorization, the image was captured with ChemiDoc MP Imaging system (Bio-Rad).

### Capillary electrophoresis with sodium dodecylsulfate (CE-SDS)

To verify the purity of Y332D, non-reduced and reduced CE-SDS were conducted. For non-reduced CD-SDS, 100 μg Y332D was mixed with 1 μl 10 kD Internal Standard and 5 μl 0.5 mol/l IAM solution, incubated at room temperature and protected from light for 30 min, and then the SDS-MW Sample buffer was added to 101 μl. The mixture was heated at 60 °C for 5 min and cooled at room temperature for 3 min. For reduced CE-SDS, 100 μg Y332D was mixed with SDS-MW Sample Buffer to make a total volume of 95 μl, and then 1 μl 10 kD Internal Standard and 5 μl β-mercaptoethanol were added. The prepared mixture was heated at 70 °C for 10 min and cooled at room temperature for 3 min. UV of migratory proteins was monitored at 214 nm using Beckman PA800 Plus.

### Surface plasmon resonance (SPR)

Protein A chip was used to immobilize Y332D by capture method, and VEGFA and TGF-β1 antigens were used as analytes to detect the kinetics and affinity of their binding to Y332D. Y332D was diluted to 2 μg/ml, and VEGFA and TGF-β1 antigens were diluted to 10 nM and 100 nM, respectively. Then, the “Biacore T200 Control Software” was run to determine the assay conditions as capture concentration of 2 μg/ml for Y332D, flow rate of 10 μl/min, and binding for 120 s. The VEGFA and TGF-β1 antigens binding parameters were 30 μl/min flow rate, 120 s binding and 600 s dissociation. The starting concentration of VEGFA antigen and Y332D were 5 nM, based on which a twofold serially dilution was applied. The starting concentration of TGF-β1 antigen and Y332D were 10 nM, based on which a twofold serially dilution was applied. The regeneration conditions were flow rate of 10 μl/min and binding for 60 s, and the regeneration reagent was 10 mM Glycine–HCl, pH 1.5. Sample detection conditions were set and 2–1 channels were selected for sample detection. After the assay was completed, the data were fitted using the “1:1 Binding” in Biacore T200 Evaluation Software, and the dissociation equilibrium constant (Kd) was calculated.

### Enzyme-linked immunosorbent assay (ELISA)

TGF-β1 (200 ng per well), VEGFA (100 ng per well) were coated in 96-well flat-bottom plates (3599, Costar) overnight at 4 °C. The next day, the plates were washed three times with PBS containing 0.05% Tween 20 and then blocked with 5% Bovine Serum Albumin (BSA, BSAS1.0, Bovogen) for 3 h. Then, serially diluted Y332D or controls (100 μl per well) were added to the plates and incubated at 37 °C for 2 h. After the plates were washed, anti-hIgG-HRP (1:5000, A80-319P, Bethyl) was added and incubated at 37 °C for 1 h. Subsequently, TMB chromogenic solution (P0209, Beyotime) was added to the washed plates, and the reaction was terminated with TMB chromogenic termination solution (P0215, Beyotime). Finally, the absorbance values were read at 450 nm.

Preparation of VEGFA-Biotin with Biotin Labeling Kit-NH2 (LK03, DoJindo): 100 µl WS buffer and 100 µg VEGFA protein solution were mixed and centrifuged at 8000 g for 10 min. The prepared mixture was added with 8 µl NH2-reactive biotin and 100 µl Reaction buffer and incubated at 37 °C for 30 min. Subsequently, the labeled VEGFA protein was washed three times with WS buffer and set aside with 100 µl WS buffer.

To detect the simultaneous binding affinity of Y332D, we performed double-antigen sandwich ELISA. TGF-β1 (200 ng per well) was coated in 96-well flat-bottom plates overnight at 4 °C. The next day, the plates were washed three times with PBS containing 0.05% Tween 20 and then blocked with 5% BSA for 3 h. Subsequently, serially diluted Y322D or controls (100 μl per well) were added to the plates and incubated at 37 °C for 2 h. Then, the plates were washed, and 100 µl prepared VEGFA-Biotin (20 ng per well) and peroxidase-conjugated streptavidin (1:5000, SA00001-0, Proteintech) were added. Finally, the plates were added with TMB chromogenic substrate and the reaction was terminated followed by the detection of absorbance values at 450 nm.

### CCK-8 assay

TGF-β1 could impede the proliferation of IL-2-dependent murine T cells [41]. CCK-8 assay was performed to explore the antagonistic effect of Y332D on the activity of TGF-β. 1 × 10^3^ CTLL-2 and HT-2 cells were seeded in 96-well plates. Then, 5 ng/ml TGF-β1 and 10^6^ pM antibodies were added. Cell viability was continuously monitored by CCK-8 reagent (10 μl per well, Dojindo) within one week after treatment.

### In vitro cytokine detection

To investigate the effect of Y332D on the alteration of TGF-β1-caused cytokine secretion during T cell activation, we conducted multi-cytokine assay using Cytometric Bead Array (CBA) Mouse Th1/Th2/Th17 Cytokine Kit (560485, BD Biosciences). Murine T cells were obtained from the isolation of splenocytes from C57BL/6 mice by Dynabeads™ Untouched™ Mouse T Cells Kit (11413D, Invitrogen). T cells (1 × 10^6^/ml) supplemented with anti-CD28 (3 μg/ml, 102116, Biolegend), TGF-β1 (5 ng/ml) and 10^6^ pM antibodies were cultured in 96 well flat-bottom plates precoated with anti-CD3 (3 μg/ml, 100302, Biolegend). After 4 days, the cellular supernatants were harvested and CBA Mouse Th1/Th2/Th17 Cytokine Kit was used to measure cytokines concentration.

### SBE4 luciferase reporter assay

3 × 10^4^ A549 or MDA-MB-231 cells were plated in 96 well flat-bottom plates and incubated at 37 °C overnight. The next day, cells were transiently transfected with 0.2 μg SBE4 luciferase reporter plasmid for each well by Lipofectamine 2000. Cells were starved 24 h post-transfection and treated with 10 ng/ml TGF-β1 and 10^6^ pM Y332D or controls for 24 h. Luminescence was detected using Bio-Lite™ Luciferase Assay System (DD1201, Vazyme).

### Western blotting

Tumor cells were lysed using RIPA buffer (P0013B, Beyotime). The supernatant was collected after centrifugation at 14,000 rpm for 15 min at 4 °C, and the total protein concentration was measured using BCA assay kit (P0010S, Beyotime). 30 µg protein sample was separated using SDS-PAGE gel (NP0321BOX, Life Tech) and transferred to a polyvinylidene fluoride membrane (ISEQ00010, Millipore). Then, the membranes were blocked with 5% BSA for 1 h and incubated with the following primary antibodies: anti-N-cadherin (1:1000, 13116, CST), anti-Vimentin (1:1000, 5741, CST), anti-GAPDH (1:1000, 5174, CST), and anti-β-Actin (1:5000, AF7018, Affinity) at 4 °C overnight. The next day, the membranes were incubated with the secondary antibodies Goat-anti-rabbit-IgG-HRP (1:2000, 7074, CST) for 1 h. SuperSignal™ West Pico PLUS (34577, Thermo Scientific) was used for visualization, and the G:BOX Chemi X system was used for signal detection.

### Transwell migration and invasion assays

To measure the motility of tumor cells, transwell migration and invasion assays were performed using 24-well Transwell apparatus containing 6.5-mm polycarbonate membrane with 8-μm pore size inserts (3422, Corning) without or with Matrigel (354234, BD Biosciences). 4T1 and EMT-6 mammary tumor cells were cultured in RPIM-1640 with 1% FBS and treated with 5 ng/ml TGF-β1 plus 10^6^ pM antibodies or untreated for 96 h. Then, about 5 × 10^4^ cells in 100 µl RPIM-1640 supplemented with 1% FBS were seeded in the upper chambers. The lower chambers were added with 600 µl of RPIM-1640 containing 10% FBS. After incubation for 24 h, the migratory and invasive cells were fixed with 4% paraformaldehyde (P0099, Beyotime) and stained with 0.1% crystal violet (C0121, Beyotime). Cell migration and invasion were evaluated by counting the migrated or invasive tumor cells in 5 random fields.

### NFAT luciferase reporter assay

NFAT is a transcription factor downstream of the VEGF/VEGFR2 pathway. HEK-293 cells overexpressing VEGFR2 were transfected with the lentiviral vectors carrying the NFAT and luciferase gene (NFAT-RE-Luci) to construct stable transfected cell lines 293-NFAT. 293-NFAT cells were seeded in the 96 well flat-bottom plates and cultured in 2% FBS-DMEM with VEGFA (20 ng/ml) and serially diluted antibodies for 6 h at 37 °C. After each well was added with 80 μl Bio-Glo™ Luciferase Assay System (PRG7941, Promega) for 15 min, the luminescence was detected.

### Luminescent cell viability assay

The inhibitory effect of Y332D on VEGFA-promoted HUVEC proliferation was measured using luminescent cell viability assay. Briefly, 5 × 10^3^ HUVEC were seeded in 96 well flat-bottom plates overnight at 37 °C. The next day, the medium in the plates was discarded and ECM premixing with VEGFA (50 ng/ml) and serially diluted antibodies or control for 30 min were added and incubated at 37 °C for 72 h. Subsequently, 100 μl Cell Counting-Lite 2.0 detection reagent (DD1101-01, Vazyme) was added to each well and incubated for 10 min at room temperature. Then, the chemiluminescence was detected.

### HUVECs tube formation assay

HUVECs were pre-cultured in ECM containing 1% FBS for 24 h. The next day, 2 × 10^4^ HUVECs were seeded in 96 well flat-bottom plates after plates were precoated with 50 μl Matrigel (354234, BD Biosciences) for 30 min at 37 °C. The cells were incubated in endothelial cell complete medium mixing with 100 ng/ml VEGFA and 10^6^ pM antibodies or control for 12 h at 37 °C. Then, HUVECs were fixed with 4% paraformaldehyde for 15 min. The images of tube-like structures were captured with inverted microscope (Olympus).

### Murine tumor models

The antitumor activities of Y332D and anti-PD-1 plus Y332D were explored in multiple murine tumor models, including H22, EMT-6, 4T1, AKT/Ras-driven murine hepatocellular carcinoma.

For H22, EMT-6 and AKT/Ras-driven murine hepatocellular carcinoma models, 8.7 mg/kg anti-PD-1 was administrated every two days by intraperitoneal injection for four times. Equivalent mole hIgG (8.7 mg/kg), anti-VEGF (8.7 mg/kg), anti-TGF-β (6 mg/kg), Y332D (10 mg/kg) were administrated on alternate days by intraperitoneal injection for six times. For 4T1 lung metastasis model, Equivalent mole hIgG (8.7 mg/kg), anti-VEGF (8.7 mg/kg), anti-TGF-β (6 mg/kg), Y332D (10 mg/kg) were administrated on alternate days by intraperitoneal injection for six times. Tumor volume (TV) of tumor-bearing mice was measured every other day or every two days. TV was calculated by the following formula: volume = length × width^2^ × 0.5. Mice were euthanatized when TV exceeded 2500 mm^3^ or the study ended.

### Subcutaneous H22 model

5 × 10^5^ H22 cells were inoculated subcutaneously in the right groin of BALB/c mice on day 0. On day 6 after inoculation, treatment was started when the TV of the tumor-bearing mice reached 50–100 mm^3^. All tumor-bearing mice were randomly divided into eight groups: Vehicle, anti-VEGF, anti-TGF-β, Y332D, anti-PD-1, anti-PD-1 plus anti-VEGF, anti-PD-1 plus anti-TGF-β, anti-PD-1 plus Y332D (6 mice for each group).

### Lung metastatic 4T1 model

2 × 10^4^ 4T1 cells were inoculated in the right mammary fat pad of BALB/c mice on day 0. Mice were anesthetized and subcutaneous tumors were removed when the TV reached 200–300 mm^3^. Then, mice were randomly divided into four groups (Vehicle, anti-VEGF, anti-TGF-β, Y332D) according to tumor volume (8 mice for each group). On day 34 after inoculation, mice were euthanized and lung tissues were collected for H&E staining.

### Orthotopic EMT‑6 model

5 × 10^5^ EMT-6 cells were inoculated in the right mammary fat pad of BALB/c mice on day 0. On day 10 after inoculation, treatment was started when the TV of the tumor-bearing mice reached 100–150 mm^3^. All tumor-bearing mice were randomly divided into six groups: Vehicle, Y332D, anti-PD-1, anti-PD-1 plus anti-VEGF, anti-PD-1 plus anti-TGF-β, anti-PD-1 plus Y332D (6 mice for each group).

### AKT/Ras-driven murine hepatocellular carcinoma model

Hydrodynamic injection was performed to establish AKT/Ras-driven murine hepatocellular carcinoma model [42]. In brief, 5 μg of the plasmid encoding myr-AKT1 and/or 25 μg of the plasmid encoding NRasV12 along with 2 μg sleeping beauty transposase were diluted in 2 ml saline (0.9% NaCl), filtered through 0.22 μm filter, and injected into the lateral tail vein of 6 to 8-week-old C57BL/6 mice in 5–7 s.

### Immunofluorescent (IF) staining

Freshly isolated tumor tissues were fixed in 10% neutral formalin for 48 h. The fixed tissues were then dehydrated, paraffin-embedded, sectioned and transferred to slides. IF staining based on tyramine signal amplification was performed according to the manufacturer's recommendations. The Multiplex Fluorescence Immunohistochemistry Kit-Four Color TSA-Rab-275 (10079100100, Panovue) was used in this assay. In addition, antibodies targeting E-cadherin (3195, CST), Vimentin (5741, CST), N-cadherin (13116, CST), α-SMA (19245, CST), CD31 (ab28364, Abcam), CD8 (98941, CST) were used in the assay. Images of IF were captured by fluorescence microscopy, previewed via Caseviewer software, and regions of interest were defined by two experienced pathologists. The quantitative analysis of IF images was conducted with ImageJ software.

### Flow cytometry analysis of tumor-infiltrating lymphocytes (TILs)

Chopped tumor tissues were enzymatically digested using the dissociation buffer supplemented with 1 mg/ml Collagenase B (11088807001, Roche) and 1 mg/ml Hyaluronidase (H3506, Sigma-Aldrich) for 1 h at 37 °C. The prepared single-cell suspensions were filtered through 40-μm nylon meshes (352340, Corning) and centrifuged at 400 g for 5 min. Then, the centrifuged cells were treated with red blood cell lysis buffer (C3702, Beyotime). Subsequently, the cells were dyed with Fixable Viability Stain 780 (565388, BD), and Fc receptors were blocked with Ultra-LEAF™ Purified anti-mouse CD16/32 (101320, BioLegend). Cells were then fluorescently stained with the following detection antibodies: α-CD45 (103132, BioLegend), α-CD3 (100206, BioLegend), α-CD8 (100706, BioLegend), α-Ki67 (151215, BioLegend), α-CD69 (104536, BioLegend), α-CD25 (102012, BioLegend), α-CD107a (121629, BioLegend), α-Granzyme-B (372214, BioLegend), α-IFN-γ (505838, BioLegend). Brilliant Stain Buffer (563794, BD Biosciences) and True-Nuclear™ Transcription Factor Buffer Set (424401, BioLegend) were used in this assay. Flow cytometry was performed using Beckman CytoFLEX LX, and the data were analyzed by FlowJo_V10.

### RNA-seq assay

At the end of H22 tumor treatment, four samples from each group were randomly selected for RNA-seq assay. The reference genome version was Mus Musculus (GRCm38/mm10). Total RNA was extracted by Trizol (Takara Bio) for cDNA library construction. Further deep sequencing was performed by Novogene (Beijing, China) via the Illumina Hiseq platform. Differentially expressed genes (DEGs) analysis was performed by R software with edgeR package and visualized by the heatmap package. DEG was identified as the gene with fold change over 2 and adjusted *p*-value less than 0.05. Immune signatures were designed based on the public lists [43]. Signature scores were defined by scaling the expression of all relevant genes within the signatures and calculating the mean value.

### Quantitative real-time polymerase chain reaction (Q-RT-PCR)

Quantitative RT-PCR was performed to measure the expression patterns of immune-related genes in the transcriptome of tumor tissues. Gene-specific primers were designed and listed in Table 1. Total RNA was extracted and reverse-transcribed. Quantitative RT-PCR was run using the 7500 Real-Time PCR System (Applied Biosystems, USA) with iQ™ SYBR^®^ Green Supermix (1708880, Bio-Rad). The assay is performed in accordance with the Minimum Information Required for Publication of Quantitative Real-Time PCR Experiments guidelines [44]. GAPDH was used as a reference control to standardize Ct values for individual genes. The relative expression level of each gene was analyzed according to 2^−ΔΔCT^ [ΔΔCt = ΔCt (test) − ΔCt (calibrator)] method [45].

**Table 1:** qPCR primers used for gene expression analysis

Gene name	Forward	Reverse
*Gzma*	TGACTGCTGCCCACTGTAACG	CGGCATCTGGTTCCTGGTTTCACA
*Prf1*	GGGACTTCAGCTTTCCAGAG	GTAGTCACATCCATGCCTTCC
*Ifng*	ATGAACGCTACACACTGCATC	CCATCCTTTTGCCAGTTCCTC
*Cxcl12*	TGCATCAGTGACGGTAAACCA	TTCTTCAGCCGTGCAACAATC
*Ptpn22*	AGCAAGCCTACAGAACGTG	TCCAGAGGTGCGTTACATATTC
*Ctla4*	ACTCATGTACCCACCGCCATA	GGGCATGGTTCTGGATCAAT
*Gapdh*	CGACTTCAACAGCAACTCCCACTCTTCC	TGGGTGGTCCAGGGTTTCTTACTCCTT

### Statistical analyses

Statistical analyses and statistical graphs were conducted by GraphPad Prism 8 software. To compare the differences between the two variables, Student's *t*-test, Welch's correction, Mann–Whitney test were applied. Student's* t*-test was applied to data with Gaussian distribution and equal variance. Welch's correction was applied to data with Gaussian distribution and heteroscedasticity. Mann–Whitney test was applied to non-normally distributed data. Mouse survival curves were calculated using the Kaplan–Meier method and compared by the log-rank test. The data were presented as mean ± standard deviation (SD). All tests in this study were two sided. *p < 0.05, **p < 0.01, ***p < 0.001, and ****p < 0.0001 indicated the significant difference.

## Results

### The characterization of bispecific antibody Y332D

**Fig. 1:**
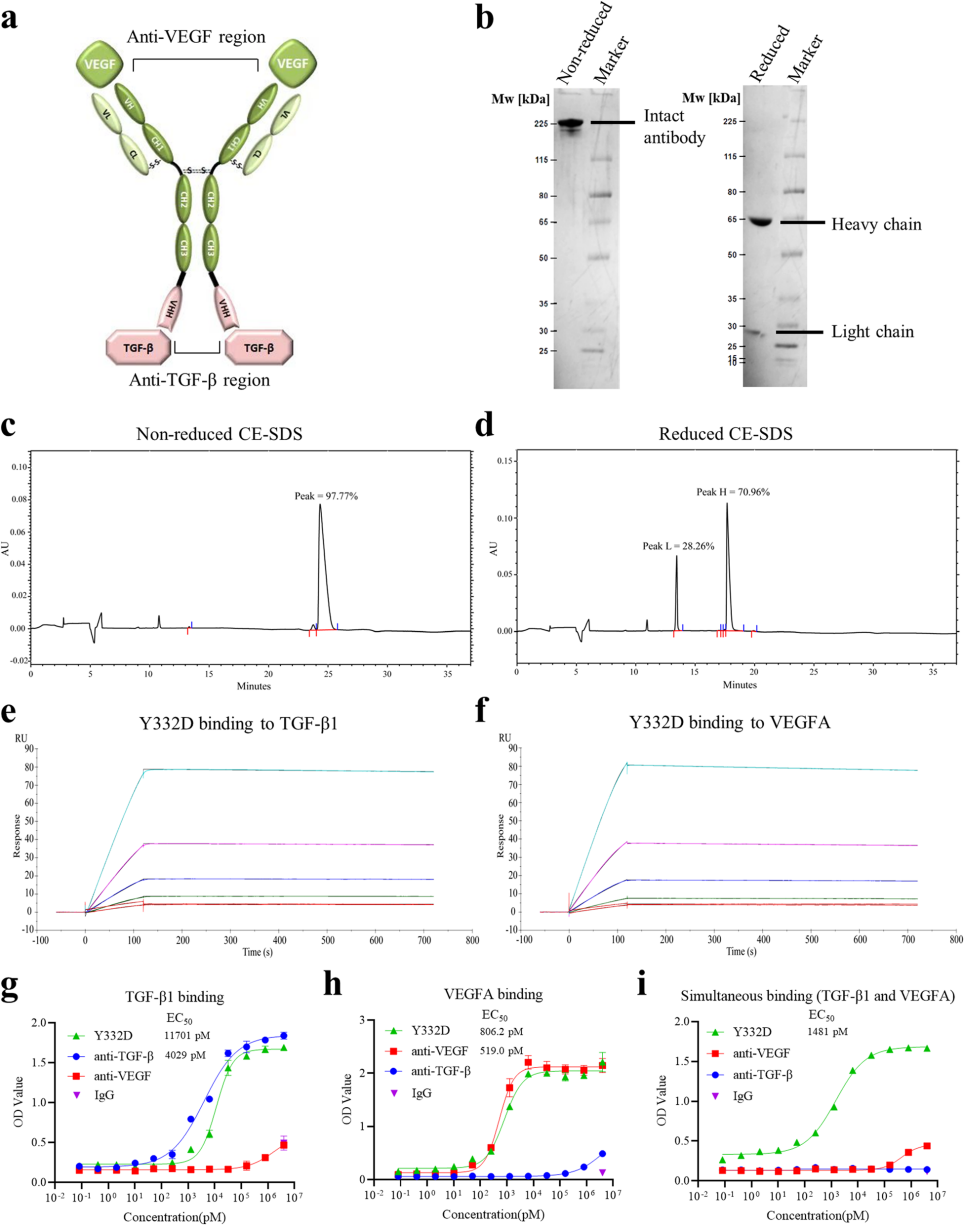
The structure characteristics of Y332D and the binding affinity of Y332D to TGF-β and VEGF. **a** Schematic representation of Y332D. Y332D is designed as a tetravalent and symmetric bispecific antibody that contains two anti-VEGF regions and two anti-TGF-β regions. The long chain of Y332D consists of five domains: VHb, CH1, CH2, CH3, VHH. The short chain of Y332D contains two domains: VLb, CL. The Fc region of Y332D is an engineered hybrid fragment: the CH2 domain is from IgG2, and the CH3 domain is from IgG1. **b** The non-reduced and reduced SDS-PAGE analysis of Y332D. Under nonreducing conditions, a single band was displayed. Two bands were observed under reducing conditions, representing the heavy and light chains. **c**, **d** The non-reduced and reduced CE-SDS analysis of Y332D. One peak was observed in the non-reduced CE-SDS, and two peaks were detected in the reduced CE-SDS. The purity of Y332D was more than 97%. **e** The results of SPR assay to detect the binding kinetics of Y332D to TGF-β1. **f** The results of SPR assay to detect the binding kinetics of Y332D to VEGFA. **g** The ELISA binding affinity of serially diluted Y332D or controls to plate-coated TGF-β1. **h** The ELISA binding affinity of serially diluted Y332D or controls to plate-coated VEGFA. **i** The simultaneous binding activity of Y332D to TGF-β1 and VEGFA by ELISA. Serially diluted Y332D or controls were incubated with plate-coated TGF-β1. Then, VEGFA-Biotin and peroxidase-conjugated streptavidin were added sequentially. Bars, SDs

Y332D is designed as a tetravalent and symmetric bispecific antibody that contains two anti-VEGF regions and two anti-TGF-β regions (Fig. 1a). The long chain of Y332D consists of five domains: VH, CH1, CH2, CH3, VHH. The short chain of Y332D contains two domains: VL, CL. The VH and VL are designed based on the sequence of human-mouse crossed anti-VEGFA monoclonal antibody G6-31 [46]. The VHH domain is derived from an anti-TGF-β single-domain antibody and isolated from an immunized *alpaca*. The Fc region of Y332D is a modified Fc fragment of human IgG1 to remove binding to FcγRs. To construct the bispecific antibody Y332D, anti-VEGF IgG was fused to VHH of anti-TGF-β single-domain antibody via a GGGGS linker.

Under nonreducing conditions, a single band (intact antibody) was displayed (Fig. 1b). Two bands (heavy and light chains) were observed under reducing conditions. Y332D was characterized for the primary structure and molecular weight (Mw) by liquid chromatograph-mass spectrometer (LC–MS). The molecular weight of intact Y332D was about 173 kDa, and the molecular weight of heavy and light chains was about 64 kDa and 23 kDa, respectively. CE-SDS assay indicated that the purity of Y332D was more than 97% (Fig. 1c, d). The purified process of Y332D is similar to that of monoclonal antibodies, both by affinity chromatography.

### Y332D demonstrated specifically binding activity for TGF-β and VEGF

To detect the binding kinetics of Y332D to mouse TGF-β1 and VEGFA, we employed the SPR assay to quantify the antigen–antibody binding ratio. The Kd for binding of Y332D to TGF-β1 was 4.631 × 10^−12^ M, and the Kd for binding of Y332D to VEGFA was 4.117 × 10^−12^ M (Fig. 1e, f). In addition, the ELISA assay was also demonstrated the binding affinity of Y332D to plate-coated TGF-β1 and VEGFA. The EC_50_ for binding of Y332D and anti-TGF-β to TGF-β1 were 11,701 pM and 4,029 pM, respectively (Fig. 1g). The EC_50_ for binding of Y332D and anti-VEGF to VEGFA were 806.2 pM and 519.0 pM, respectively (Fig. 1h). Compared to parent anti-TGF-β and anti-VEGF mAbs, Y332D exhibited a slight increase in EC_50._ In the double-antigen sandwich ELISA assay, Y332D could capture precoated TGF-β1 and VEGFA simultaneously, and the EC_50_ was 1481 pM (Fig. 1i). ELISA assay revealed that Y332D had comparable affinities with the parent antibodies, which provides theoretical support for the dual target blocking effect of Y332D.

### Y332D antagonized TGF-β-induced immunosuppression, activation of TGF-β signaling pathway and epithelial-mesenchymal transition (EMT)

**Fig. 2:**
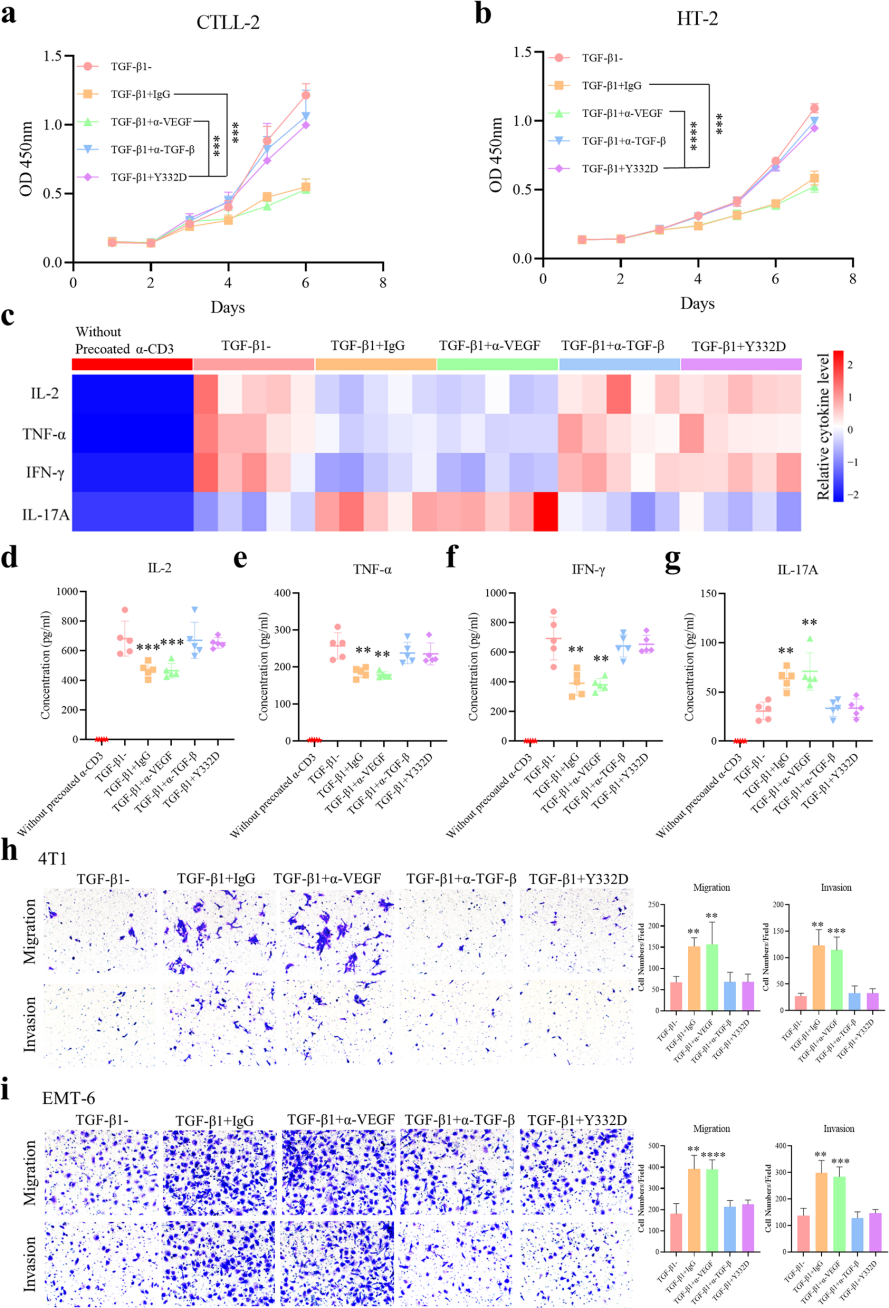
Y332D counteracted TGF-β1-induced inhibition of T cell proliferation and activation as well as epithelial-mesenchymal transition (EMT). **a**, **b** CCK-8 assays were performed to show the antagonistic effect of Y332D on TGF-β1-hampered proliferation in T cells. 1 × 10^3^ CTLL-2 and HT-2 cells were seeded in 96-well plates. Then, 5 ng/ml TGF-β1 plus 10^6^ pM antibodies or control were added. Cell viability was continuously monitored by CCK-8 reagent. **c**–**g** Multi-cytokine assay was performed to analyze the effect of Y332D on the alteration of TGF-β1-caused cytokine secretion during T cell activation. Murine T cells were obtained from the isolation of splenocytes from C57BL/6 mice. T cells (1 × 10^6^/ml) supplemented with anti-CD28 (3 μg/ml), TGF-β1 (5 ng/ml) and 10^6^ pM antibodies or control were cultured in 96 well flat-bottom plates precoated with anti-CD3 (3 μg/ml). After 4 days, the cellular supernatants were harvested to measure cytokines concentration. **h**, **i** Transwell migration/invasion assays were performed to demonstrate the antagonistic effect of Y332D on TGF-β-enhanced tumor cell motility. 4T1 and EMT-6 mammary tumor cells were cultured in RPIM-1640 with 1% FBS and treated with 5 ng/ml TGF-β1 plus 10^6^ pM antibodies or untreated for 96 h. Then, about 5 × 10^4^ cells in 100 µl RPIM-1640 supplemented with 1%FBS were seeded in the upper chambers. The lower chambers were added with 600 µl of RPIM-1640 containing 10% FBS. After incubation for 24 h, the migratory and invasive cells were fixed with 4% paraformaldehyde and stained with 0.1% crystal violet. Bars, SDs; **p < 0.01, ***p < 0.001, and ****p < 0.0001 denote the significant difference relative to Y332D treatment. α-TGF-β: anti-TGF-β, α-VEGF: anti-VEGF

TGF-β is a vital immunosuppressive cytokine. We investigated the blocking effect of Y332D on TGF-β-mediated immunosuppression. CCK-8 assay showed that TGF-β1 impeded the proliferation of IL-2-dependent murine T cell lines CTLL-2 and HT-2. Y332D reversed TGF-β1-hampered proliferation in T cells (Fig. 2a, b). Multi-cytokine assay indicated that exogenous TGF-β1 reduced the levels of multiple cytokines (IL-2, TNF-α, IFN-γ) but increased the release of IL-17A. Y332D reversed TGF-β1-caused alteration in cytokine secretion during T cell activation (Fig. 2c–g).

Canonical TGF-β signaling promotes Smad2/3 phosphorylation and transformation from epithelial phenotype to mesenchymal phenotype, which enhances the invasive capacity of tumor cells. Research revealed that TGF-β mediates the transcription of Smad-Binding Element-containing luciferase reporter construct, SBE4-Luc [47]. Therefore, SBE4 luciferase reporter assay was performed to test the blocking capability of Y332D on TGF-β/Smad pathway. The results showed that Y332D remarkedly blocked TGF-β1 signaling in A549 and MDA-MB-231 cells (Additional file 1: Fig. S1a, b). Also, Y332D significantly antagonized TGF-β1-regulated EMT in A549 and MDA-MB-231 cells. The expression of mesenchymal markers N-cadherin and Vimentin were decreased (Additional file 1: Fig. S1c, d). In addition, we also measured the antagonistic effect of Y332D on TGF-β-enhanced tumor cell motility by transwell migration/invasion assays. The results showed that 4T1 and EMT-6 mammary tumor cells treated with TGF-β1 displayed increased migratory and invasive capacity. Tumor cell migration and invasion were impaired by Y332D (Fig. 2h, i).

### Y332D blocked the activity of VEGF/VEGFR signaling pathway

**Fig. 3:**
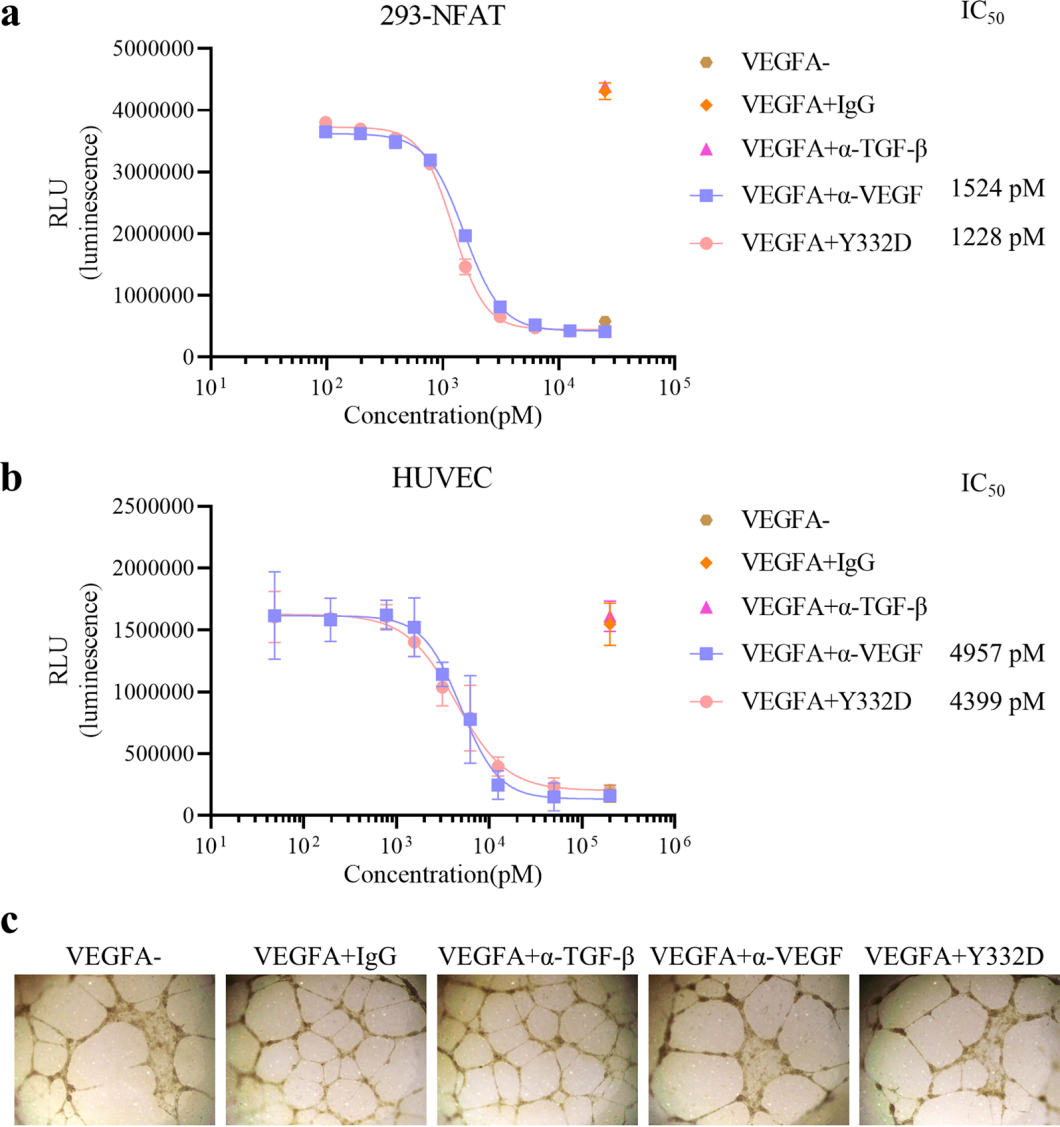
The antagonistic effect of Y332D on the activation of VEGF/VEGFR pathway, VEGFA-induced proliferation and tube formation in HUVECs. **a** NFAT-luciferase reporter assay was performed to show the blockade effect of Y332D on VEGF/VEGFR pathway. HEK-293 cells overexpressing VEGFR2 were transfected with the lentiviral vectors carrying the NFAT and luciferase gene (NFAT-RE-Luci) to construct stable transfected cell lines 293-NFAT. 293-NFAT cells were cultured in 2% FBS-DMEM with VEGFA (20 ng/ml) and serially diluted Y332D or controls for 6 h. Then, the luminescence was detected. **b** Luminescent cell viability assay was performed to measure the inhibitory effect of Y332D on VEGFA-induced HUVEC proliferation. 5 × 10^3^ HUVECs were seeded in 96-well plates overnight at 37 °C. Then, the medium was replaced with endothelial cell basal medium mixed with VEGFA (50 ng/ml) and serially diluted Y332D or controls. Cell viability was detected after incubation at 37 °C for 72 h. **c** Tube formation assay was performed to show the inhibitory effect of Y332D on VEGFA-induced vessel-like tube formation. 2 × 10^4^ HUVECs were seeded in 96 well flat-bottom plates after plates were precoated with 50 μl Matrigel for 30 min at 37 °C. The cells were incubated in endothelial cell complete medium mixing with 100 ng/ml VEGFA and 10^6^ pM antibodies or control for 12 h at 37 °C. Then, HUVECs were fixed with 4% paraformaldehyde for 15 min. The images of tube-like structures were captured with inverted microscope. Bars, SDs; α-TGF-β: anti-TGF-β, α-VEGF: anti-VEGF

VEGF secreted by tumor cells promotes tumor vascularization by binding to VEGFR and subsequently causing the activation of downstream signaling cascades [48]. We investigated the blocking capability of Y332D on VEGF/ VEGFR pathway using HEK-293 cells overexpressing VEGFR and transfected with NFAT-luc. The results of NFAT luciferase reporter assay showed that VEGFA promoted the transcription activity of NFAT, and this effect was blocked by Y332D (IC_50_ = 1228 pM) or anti-VEGF (IC_50_ = 1524 pM) (Fig. 3a).

As a highly specific pro-vascular endothelial cell growth factor, VEGFA has been shown to promote endothelial cell survival and proliferation as well as play a crucial physiological role in angiogenesis, maintenance, and enhancement of vascular permeability [49, 50]. We validated the in vitro inhibitory properties of Y332D to VEGF-mediated effects on HUVEC. The results showed that Y332D and anti-VEGF effectively inhibited VEGFA-stimulated HUVEC proliferation, and the IC_50_ values were 4399 pM and 4957 pM, respectively (Fig. 3b). Also, VEGF-induced vessel-like tube formation was significantly reduced in the presence of Y332D or anti-VEGF (Fig. 3c).

### Y332D inhibited tumor growth and metastasis in murine tumor models, and the combination of Y332D with PD-1 blockade demonstrated synergistic antitumor effect

**Fig. 4:**
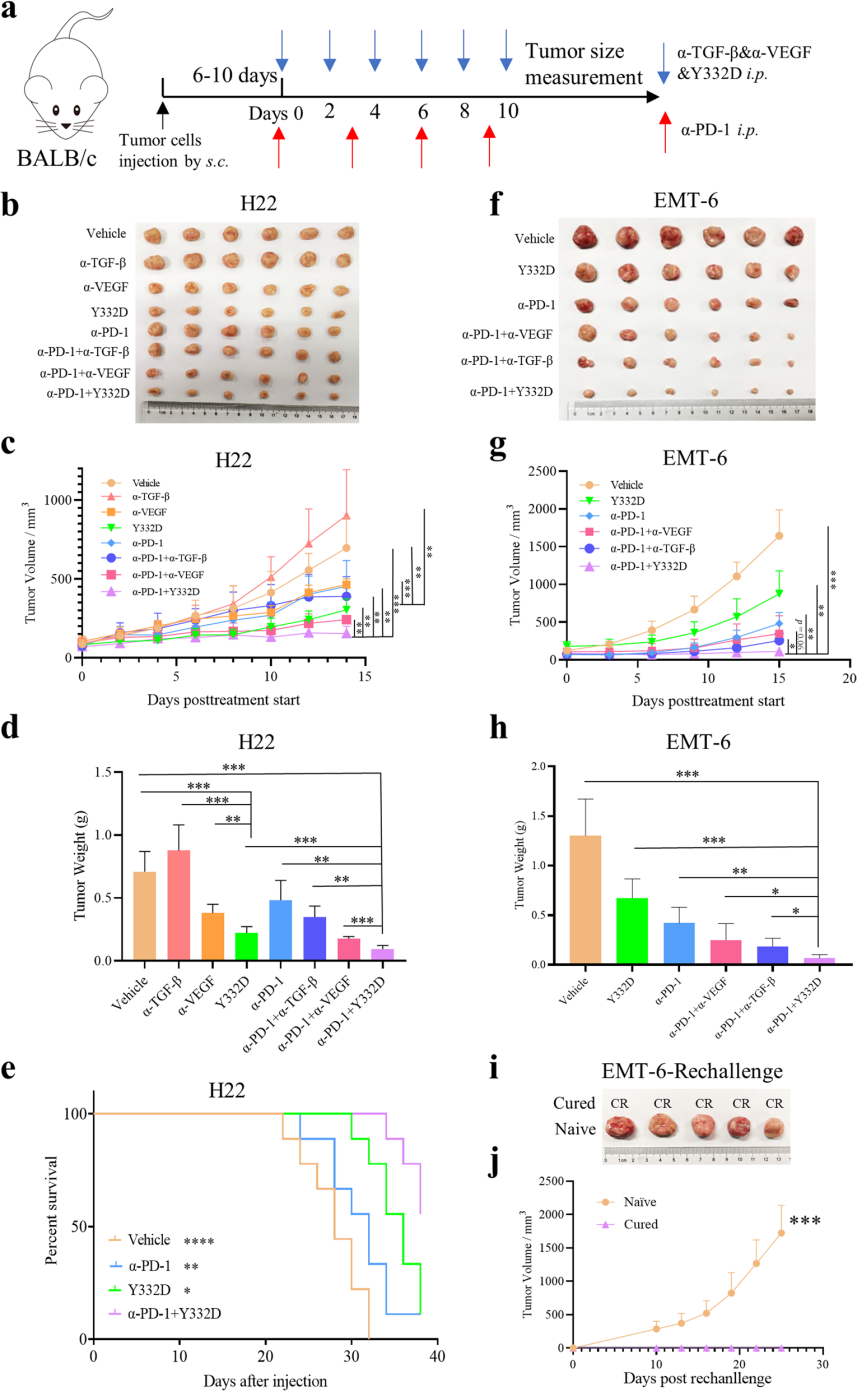
Y332D inhibited tumor growth in murine tumor models, and the combination of Y332D and PD-1 blockade demonstrated synergistic antitumor effects. Tumor volume (TV) of tumor-bearing mice was measured every other day or every two days. Mice were euthanatized when TV exceeded 2500 mm^3^ or the study ended. **a** Model establishment and treatment schedule of H22 and EMT-6 tumor models. 8.7 mg/kg α-PD-1 were administrated every two days by intraperitoneal injection for four times. Equivalent mole hIgG (8.7 mg/kg), α-VEGF (8.7 mg/kg), α-TGF-β (6 mg/kg), Y332D (10 mg/kg) were administrated on alternate days by intraperitoneal injection for six times. **b**–**d** 5 × 10^5^ H22 cells were inoculated subcutaneously in the right groin of BALB/c mice on day 0. Start of treatment on day 6. The representative tumors image, tumor growth curve, tumor weight of H22-bearing mice receiving α-PD-1 plus Y332D or controls treatment were shown. **e** The overall survival curves of H22-bearing mice receiving α-PD-1 plus Y332D or controls treatment were shown. **f**–**h** 5 × 10^5^ EMT-6 cells were inoculated in the right mammary fat pad of BALB/c mice on day 0. Start of treatment on day 10. The representative tumors image, tumor growth curve and tumor weight of EMT-6-bearing mice receiving α-PD-1 plus Y332D or controls treatment were shown. **i**, **j** The representative image and tumor growth curve of EMT-6 tumors in the rechallenge assay were shown. *p < 0.05, **p < 0.01, ***p < 0.001, and ****p < 0.0001 denote the significant difference relative to Y332D or Y332D plus anti-PD-1 treatment. CR: complete regression. Bars, SDs; α-PD-1: anti-PD-1, α-TGF-β: anti-TGF-β, α-VEGF: anti-VEGF

We compared the antitumor activity of Y332D with vehicle, anti-VEGF, anti-TGF-β in multiple murine syngeneic tumor models including H22 and 4T1. In murine H22 hepatocarcinoma model, anti-VEGF exhibited partial antitumor activity while anti-TGF-β did not inhibit tumor growth. Y332D demonstrated superior antitumor efficacy to anti-VEGF (Fig. 4b–d). We also investigated the metastasis inhibitory activity of Y332D in lung metastatic 4T1 murine model. Y332D significantly superior to anti-VEGF and anti-TGF-β in reducing the number of 4T1 tumor nodules in lung tissue (Additional file 1: Fig. S2a, b).

**Fig. 5:**
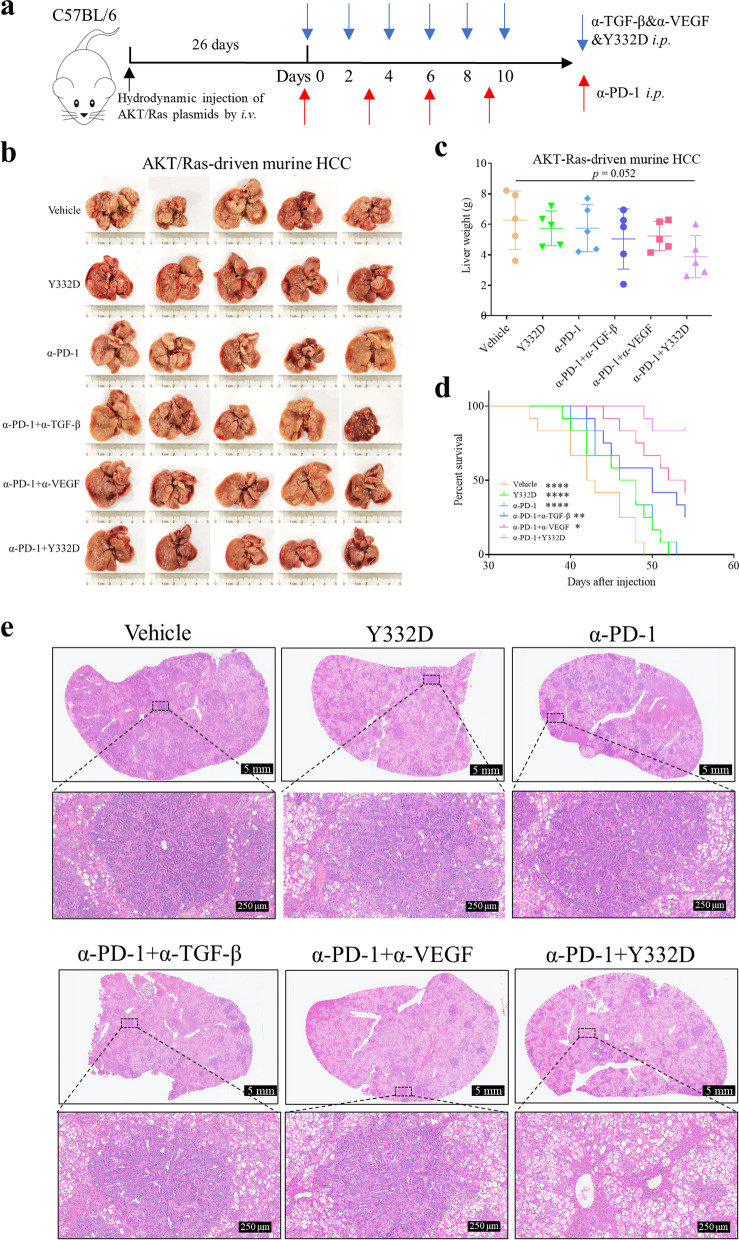
The combination of Y332D and PD-1 blockade demonstrated synergistic antitumor efficacy in AKT/Ras-driven murine hepatocellular carcinoma tumor model. **a** Model establishment and treatment schedule of AKT/Ras-driven murine hepatocellular carcinoma tumor model. The plasmid encoding myr-AKT1 and/or NRasV12 along with sleeping beauty transposase were injected into the lateral tail vein of C57BL/6 mice by hydrodynamic injection on day 0. Treatment was started on day 26 (5 mice for each group). All tumor-bearing mice were randomly divided into six groups (Vehicle, Y332D, α-PD-1, α-PD-1 plus α-VEGF, α-PD-1 plus α-TGF-β, α-PD-1 plus Y332D). 8.7 mg/kg α-PD-1 were administrated every two days by intraperitoneal injection for four times. Equivalent mole hIgG (8.7 mg/kg), α-VEGF (8.7 mg/kg), α-TGF-β (6 mg/kg), Y332D (10 mg/kg) were administrated on alternate days by intraperitoneal injection for six times. 40 days after injection, the mice were euthanized, and the liver tissues were collected. **b**, **c** The representative liver tumor images and liver weight of AKT/Ras-driven murine hepatocellular carcinoma mice receiving α-PD-1 plus Y332D or controls treatment were shown. **d** The overall survival curves of AKT/Ras-driven murine hepatocellular carcinoma mice receiving α-PD-1 plus Y332D or controls treatment were shown. **e** The representative H&E staining images of liver tissues of mice receiving the treatment of combination therapies or controls. The size of the scale bar in the immunofluorescence images refer to 5 mm or 250 μm. *p < 0.05, **p < 0.01, and ****p < 0.0001 denote the significant difference relative to Y332D plus anti-PD-1 treatment. Bars, SDs. α-PD-1: anti-PD-1, α-TGF-β: anti-TGF-β, α-VEGF: anti-VEGF

In addition, previous studies have shown synergistic effects of anti-PD-1 plus anti-VEGF or anti-PD-1 plus anti-TGF-β in murine tumor models. Here, we observed that anti-PD-1 plus Y332D demonstrated the most potent antitumor efficacy among all treatments in murine H22 (Fig. 4b–d), EMT-6 (Fig. 4f–h) and AKT/Ras-driven murine hepatocellular carcinoma tumor models (Fig. 5b, e) without increasing the physical burden (Additional file 1: Fig. S3a–c). In the EMT-6 rechallenge assay, anti-PD-1 plus Y332D inhibited tumor regrowth and provided durable immune protection (Fig. 4i, j). Additionally, we also explored the effect of anti-PD-1 plus Y332D on the survival of tumor-bearing mice. In H22 and AKT/Ras-driven murine hepatocellular carcinoma tumor models, anti-PD-1 plus Y332D significantly prolonged the survival time of mice (Fig. 4e and Fig. 5d). We also weighed the liver tissues of AKT/Ras-driven murine hepatocellular carcinoma tumor model and found no significant differences between the groups (Fig. 5c).

### Y332D reversed EMT of cancer cells, inhibited the activity of CAFs and reduced tumor angiogenesis in H22 tumor model

**Fig. 6:**
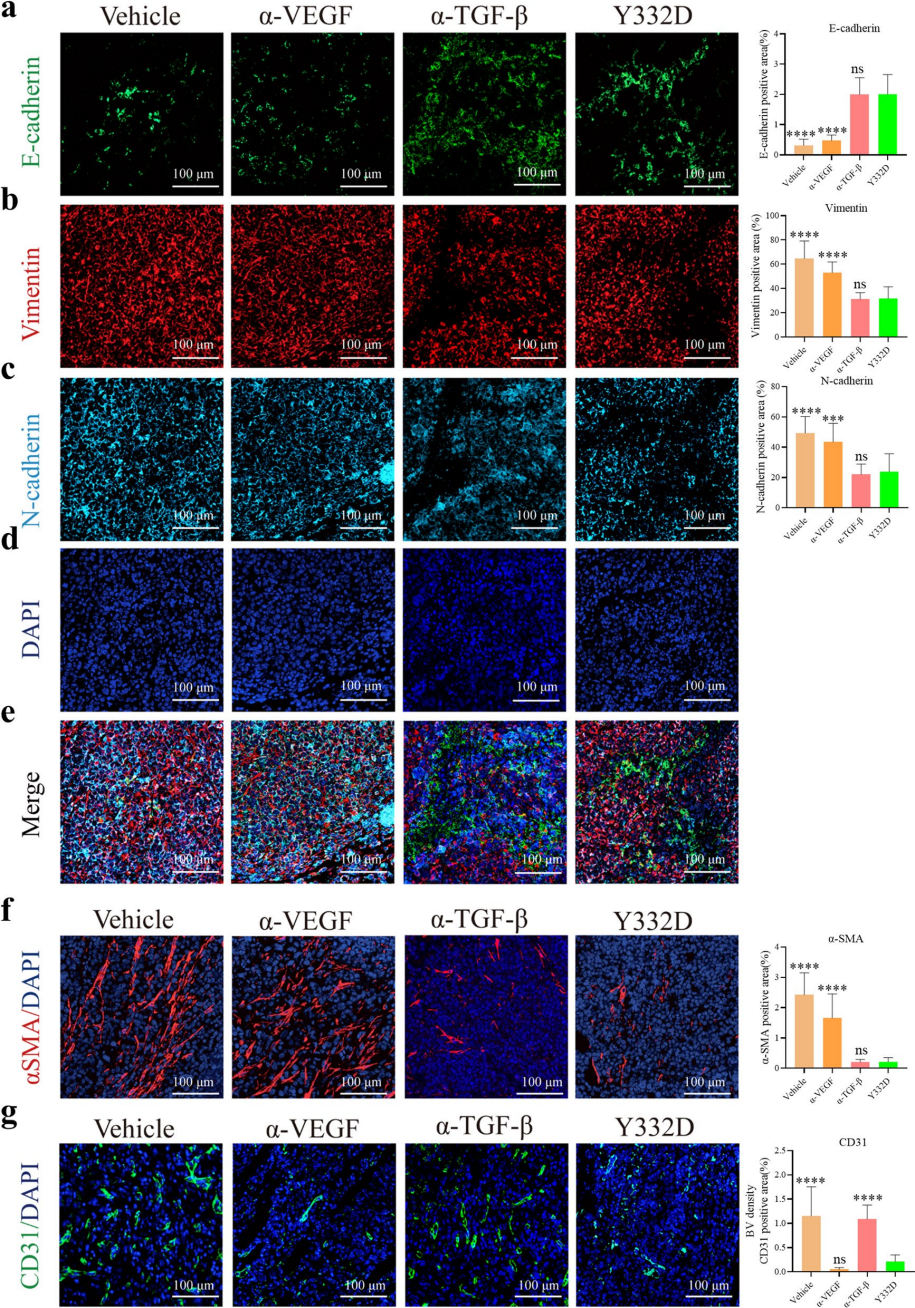
Immunofluorescence staining to measure the status of epithelial-mesenchymal transition (EMT) of cancer cells, carcinoma-associated fibroblast (CAF) and tumor angiogenesis in H22 tumor model. The representative images and quantitative analysis of **a**–**e** EMT-related markers, including anti-E-cadherin staining, anti-Vimentin staining, anti-N-cadherin staining, **f** CAF marker, anti-α-SMA staining, **g** Anti-CD31 staining. The size of the scale bar in the immunofluorescence images refer to 100 μm. Bars, SDs; ***p < 0.001, and ****p < 0.0001 denote the significant difference relative to Y332D treatment. ns: not significant, α-TGF-β: anti-TGF-β, α-VEGF: anti-VEGF

To validate the in vivo effect of the anti-TGF-β moiety of Y332D, we investigated the EMT-related markers E-cadherin, Vimentin, N-cadherin and the CAF marker α-SMA by IF staining assay in H22 tumor model. The results showed that Y332D and anti-TGF-β significantly decreased the expression of Vimentin, N-cadherin, α-SMA but increased the expression of E-cadherin relative to vehicle and anti-VEGF (Fig. 6a–f). Moreover, anti-CD31 IF staining was performed to identify the in vivo activity of the anti-VEGF moiety of Y332D. Compared with vehicle and anti-TGF-β, Y332D significantly reduced the expression of CD31. A similar result was observed in anti-VEGF-treated tumors (Fig. 6g). Our data demonstrated that Y332D exhibited combined antitumor activities by simultaneously inhibiting EMT of tumor cells, reducing CAFs and tumor angiogenesis.

### Y332D plus anti-PD-1 therapy promoted the infiltration of T cells and enhanced the cytotoxicity of T cells in H22 tumor models

**Fig. 7:**
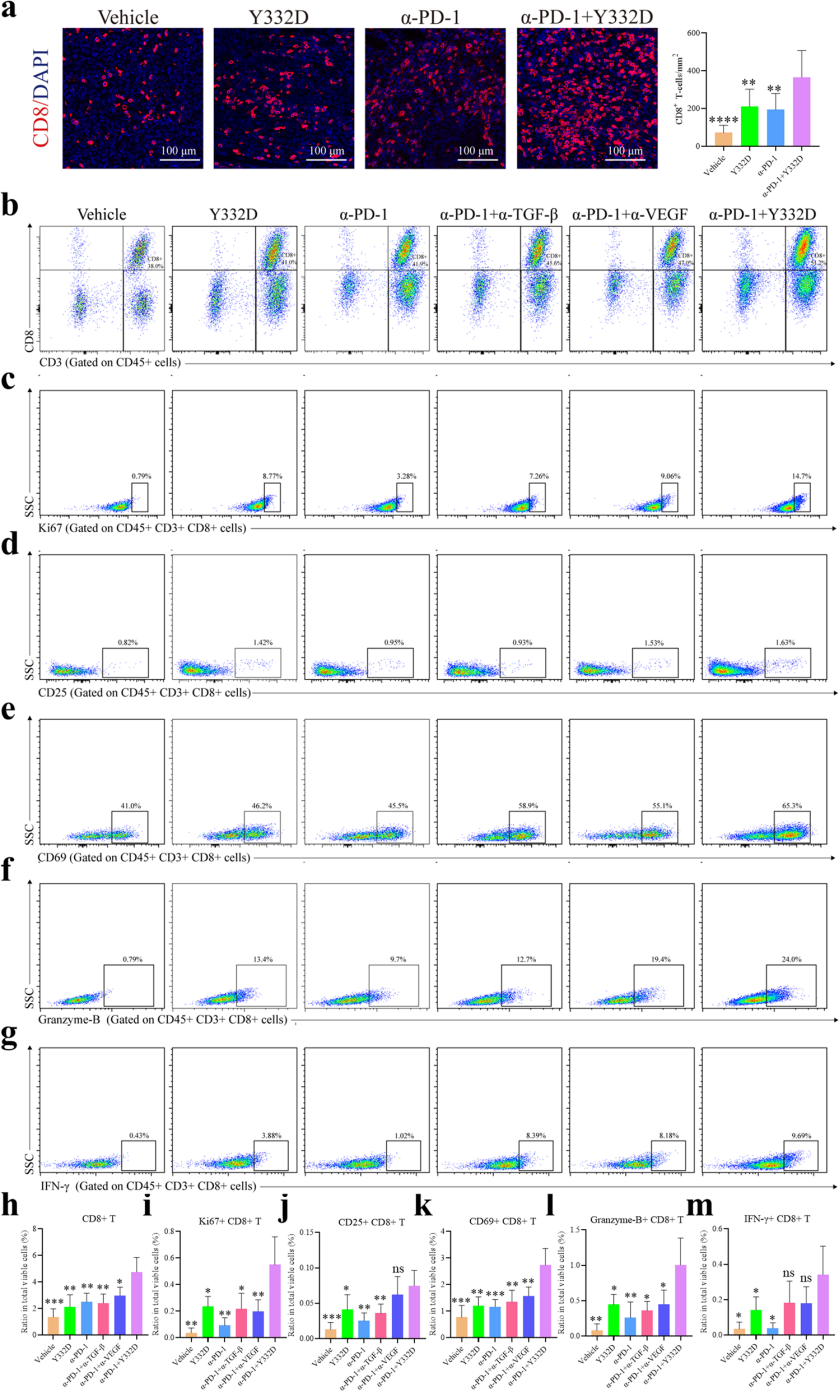
Immunofluorescence staining and flow cytometry assay to analyze tumor-infiltrating lymphocytes in H22 tumor model. Mice were euthanatized when the study ended. The harvested tumor tissues were subjected to immunofluorescence and flow cytometry. **a** The representative immunofluorescence images and quantitative analysis of tumor-infiltrating CD8^+^ T cells. Harvested tumor tissues were dissociated with Collagenase B and hyaluronidase to prepare single-cell suspensions. Then, the cells were fluorescently stained with the detection antibodies. The representative images and quantitative analysis of tumor-infiltrating **b**, **h** CD8^+^ T cells, **c**, **i** Ki67^+^CD8^+^ T cells, **d**, **j** CD25^+^CD8^+^ T cells, **e**, **k** CD69^+^CD8^+^ T cells, **f**, **l** Granzyme B^+^CD8^+^ T cells, **g**, **m** IFN-γ^+^CD8^+^ T cells. The proportion of tumor-infiltrating lymphocytes in the total live cells was calculated. The size of the scale bar in the immunofluorescence images refer to 100 μm. Bars, SDs; *p < 0.05, **p < 0.01, ***p < 0.001, and ****p < 0.0001 denote the significant difference relative to combination treatment. ns: not significant, α-PD-1: anti-PD-1

As previously described, TGF-β and VEGF disrupt immune cell function and prevent T cell infiltration toward the tumor site [51]. In H22 murine tumor model, we investigated the effect of combination therapy on T cell infiltration by IF staining. IF staining results revealed a significantly increased frequency in TILs in the combination therapy group (Fig. 7a).

We next performed flow cytometry assay to explore the activity of TILs in H22 tumor model and found that Y332D plus anti-PD-1 therapy significantly upregulated the density and function of TILs compared to other groups. The results of flow cytometry showed that the quantity of tumor-infiltrating lymphocytes, CD3^+^ T cells and CD8^+^ T cells were significantly increased in Y332D plus anti-PD-1 therapy group (Additional file 1: Fig. S4a, b) (Fig. 7b, h). Besides, the increase in proliferating CD3^+^ T cells and CD8^+^ T cells (Ki67^+^ CD3^+^ and Ki67^+^ CD8^+^), activated CD3^+^ T cells (CD69^+^ CD3^+^), activated CD8^+^ T cells (CD25^+^ CD8^+^ and CD69^+^ CD8^+^), cytotoxic CD3^+^ T cells (CD107a^+^ CD3^+^, Granzyme B^+^ CD3^+^) and cytotoxic CD8^+^ T cells (Granzyme B^+^ CD8^+^, IFN-γ^+^ CD8^+^) in the combination therapy group implied that anti-PD-1 plus Y332D therapy significantly induced activation of cytotoxic T-cells and enhanced the tumor-killing ability of TILs (Fig. 7c–g, i–m) (Additional file 1: Fig. S4c–f).

### Y332D plus anti-PD-1 therapy enhanced the gene expression and pathways enrichment related to antitumor immunity

**Fig. 8:**
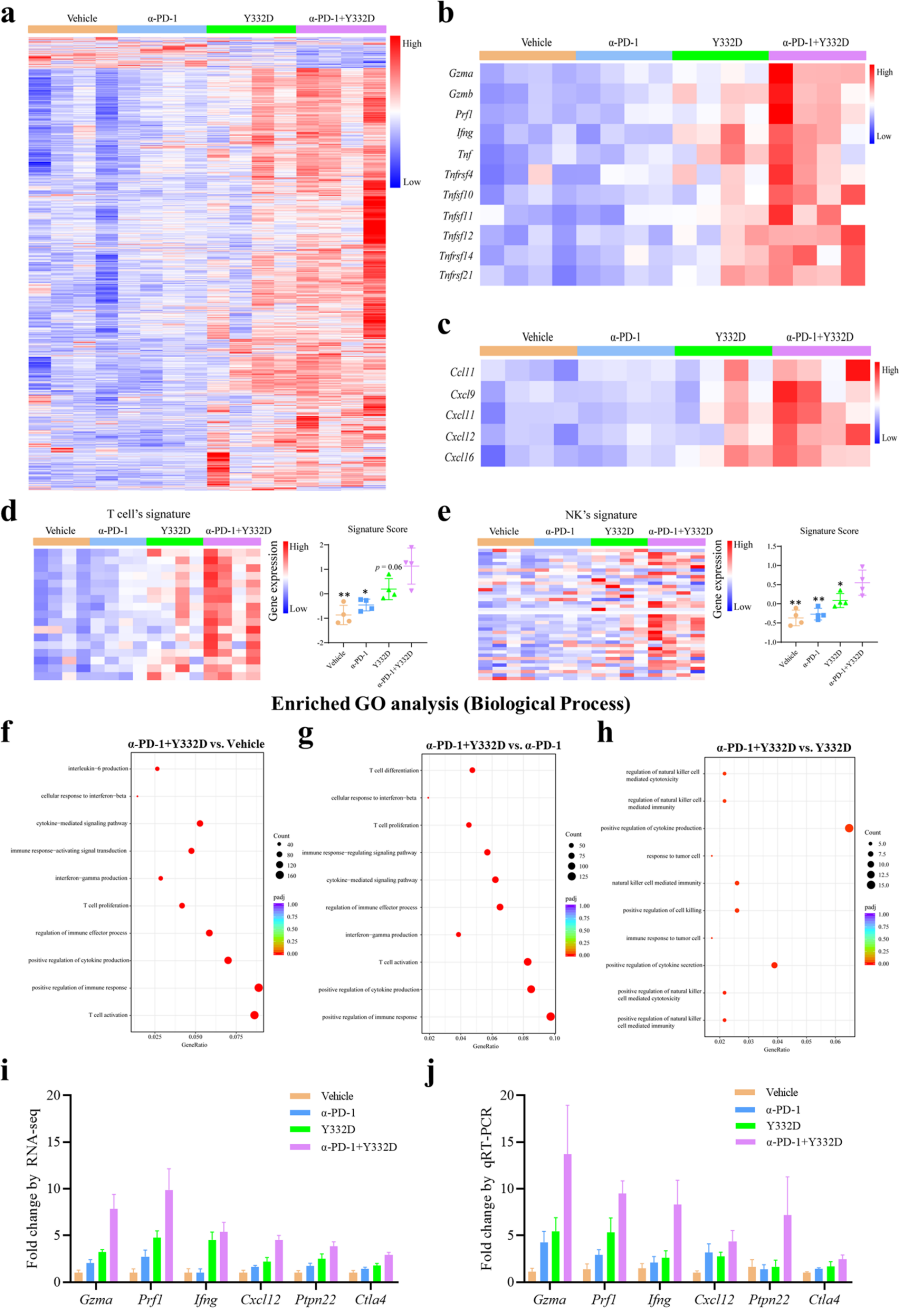
RNA-seq assay to explore the immune profile of H22 tumors after different treatments. **a** Heat map to represent the differentially expressed genes among four groups. **b** Heat map to represent the expression levels of cytotoxicity-related genes (*Gzma, Gzmb, Prf1, Ifng, Tnf,* etc.). **c** Heat map to represent the expression levels of chemokines (*Ccl11, Cxcl9, Cxcl11, Cxcl12, Cxcl16*). **d**, **e** Heat map to represent the expression levels of signature genes in T cells and NK cells, and signature scores were calculated to quantify. **f–h** The top 10 significantly enriched immune-related Gene Ontology (GO) terms (α-PD-1 + Y332D vs. Vehicle; α-PD-1 + Y332D vs. α-PD-1; α-PD-1 + Y332D vs. Y332D). **i**, **j** Quantitative RT-PCR validation of selected genes identified by RNA-seq. Bars, SDs; *p < 0.05 and **p < 0.01 denote the significant difference relative to combination treatment. α-PD-1: anti-PD-1

The interaction of chemokines and chemokine receptors recruits different immune cells into the tumor microenvironment [52]. The inflammatory/cytotoxic effector genes such as *Gzma*, *Prf1*, and *Ifng* are validated to enhance the cytotoxic function of lymphocytes [53]. We performed RNA-seq assay to explore the effect of the combination therapy on immune-related genes profile in H22 tumor model. Analysis of all differentially expressed genes revealed significantly higher expression levels of cytotoxicity-related genes (*Gzma, Gzmb, Prf1, Ifng, Tnf,* etc.) and chemokines (*Ccl11, Cxcl9, Cxcl11, Cxcl12, Cxcl16*) in Y332D plus anti-PD-1 therapy group (Fig. 8a–c). Moreover, we analyzed the altered tumor microenvironment of tumor tissues using multiple immune cell signatures. The results showed that remarkedly higher signature scores of T and NK cell signatures were observed in the Y332D plus anti-PD-1 therapy group (Fig. 8d, e).

In addition, GO enrichment analysis showed that multiple immune-related pathways significantly enriched in the combined group compared to the other three groups, including cytokine production, secretion and signaling pathway, cellular response to IFN-β, immune response-activating signal transduction, IFN-γ production, T cell proliferation, differentiation and activation, regulation of immune effector process, positive regulation of immune response and related signaling pathway, positive regulation of NK cell-mediated cytotoxicity and immunity, immune response to tumor cell, positive regulation of cell killing (Fig. 8f–h). The results of KEGG and GSEA enrichment analysis showed that multiple immunity-related signaling pathways significantly enriched in the combination of Y332D with anti-PD-1 therapy group (Additional file 1: Fig. S5a–f). The analysis of transcriptomic data suggested that the combination of Y332D with PD-1 blockade showed potent antitumor immunity by enhancing multiple steps in the tumor-immune cycle. We also performed quantitative RT-PCR using gene-specific primers to verify the expression patterns of multiple immune-related genes and found that they are consistent with observations from RNA-seq (Fig. 8i, j).

## Discussion

**Fig. 9:**
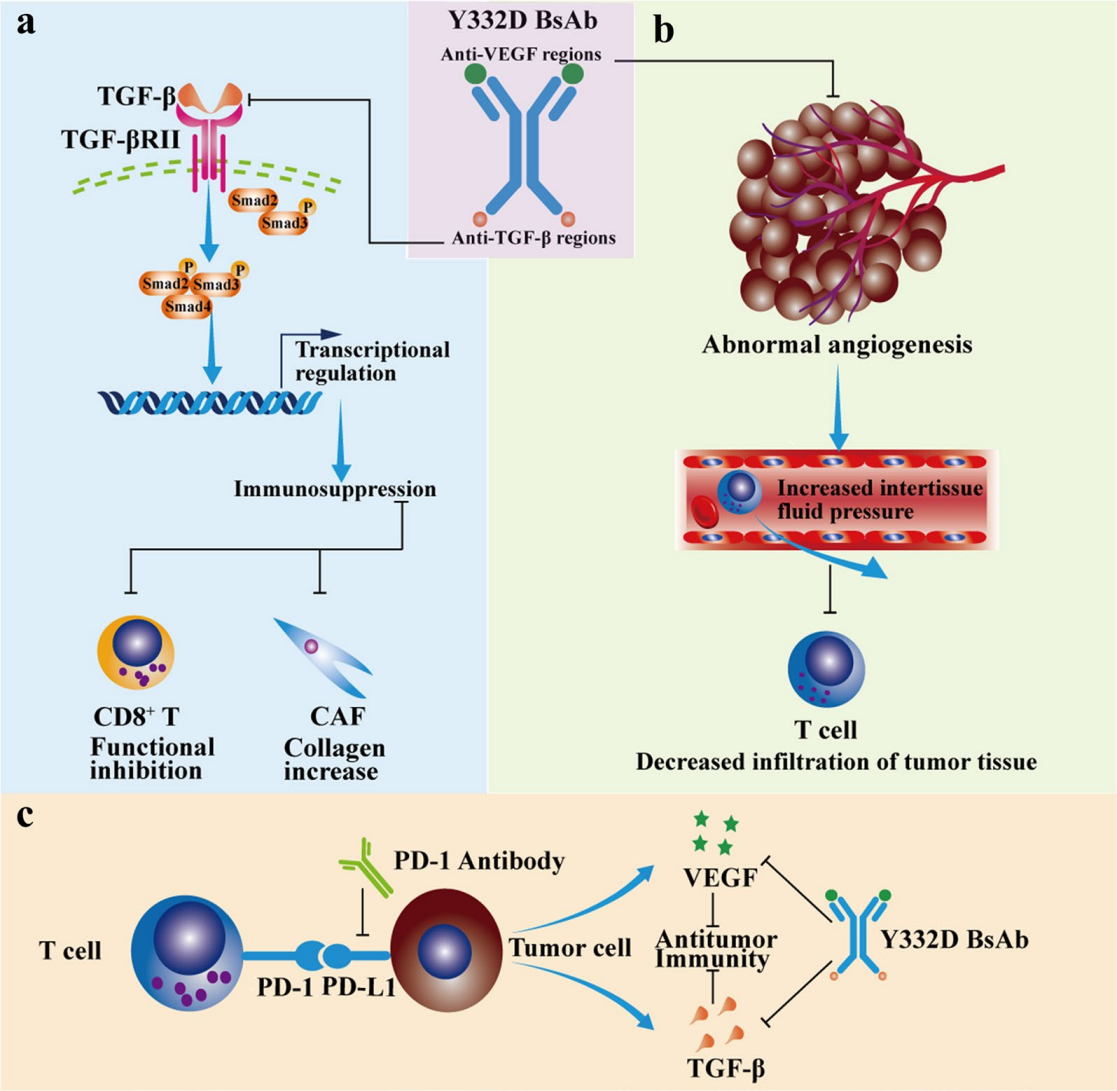
Schematic diagram demonstrating the synergistic antitumor immune effect of Y332D plus PD-1 blockade. **a** Y332D restored the cytotoxic effects of TGF-β-suppressed CD8^+^ T cells and inhibited TGF-β-mediated collagen production in cancer-associated fibroblasts (CAF) cells. **b** Y332D promoted T cells infiltration through vascular normalization. **c** Y332D in combination with PD-1 blockade synergistically enhanced antitumor immunity

The emergence of immunotherapy has changed the conventional treatment paradigm, but the response rate of most patients to PD-1/PD-L1 antibodies is unsatisfactory. How to improve the efficiency of immunotherapy has become an urgent issue to be addressed. Most ‘immune-cold’ tumors are resistant to PD-1/PD-L1 antibodies [54]. The diversity of immune evasion mechanisms remains a key obstacle in turning nonresponsive ‘cold’ tumors into responsive ‘hot’ tumors [54, 55]. TGF-β and VEGF impede immune cell infiltration by promoting peritumoral collagen production and tumor angiogenesis, resulting in a cold tumor immunophenotype [15, 30]. Therefore, we developed Y332D, a bispecific antibody targeting both TGF-β and VEGF, to potentially increase the sensitivity of PD-1 antibodies by promoting the transition from ‘cold’ tumors to ‘hot’ ones (Fig. 9a–c).

The bispecific antibody being developed must maintain the key properties of the parent molecules. According to the results of SPR and ELISA affinity assays, Y332D maintained its affinity for both TGF-β and VEGF, with binding affinities were comparable to or slightly weaker than those of the parent molecules. Functionally, Y332D acted as a TGF-β blocker and antagonized the biological activities of TGF-β just as efficiently as the parent anti-TGF-β antibody. According to in vitro data, Y332D antagonized TGF-β-induced inhibition of T cell proliferation and activation. In addition, Y332D blocked TGF-β signaling and reversed TGF-β-induced EMT in tumor cells. Similarly, Y332D also retained the functional activities of the parent anti-VEGF antibody. In vitro data showed that Y332D blocked the activation of VEGF/VEGFR pathway as well as HUVEC proliferation and tube formation.

In H22 murine tumor model, the anticancer activity of Y332D was superior to anti-TGF-β or anti-VEGF therapy. We verified the effect of Y332D on tumor cells and tumor microenvironment in H22 tumor tissues by IF staining and found that Y332D remarkedly reduced the expression of Vimentin, N-cadherin, α-SMA, CD31 and increased the expression of E-cadherin. Y332D also almost completely inhibited metastatic nodule formation in the lung metastasis 4T1 model.

In addition, we noted that in numerous mouse tumor models, the combination of Y332D and anti-PD-1 antibody demonstrated noticeably stronger and more durable antitumor activity than anti-PD-1 antibody or Y332D alone. Given that all three targets are immune-related, we investigated the impact of combination therapy on the tumor microenvironment using IF, flow cytometry, and RNA-seq assay in H22 tumor model. The results indicated that combination therapy substantially increased the frequency and quality of TILs, and various biological processes related to antitumor immunity were significantly enriched. Our findings demonstrated that the combination strategy of Y332D and anti-PD-1 antibody successfully overcame treatment resistance due to immune exclusion.

Previous studies have shown that the gene expression of TGF-β signature, which was classified as highly activated, almost exactly overlaps with the gene expression profile of the immune exhaustion classification as well as the upregulation of *VEGFA* gene expression [32]. This provides some theoretical support for combining the anti-PD-1 antibody with Y332D. In addition, the combination of VEGF-blocking and TGF-β-blocking drugs was reported to exert synergistic inhibitory effects on tumor growth in B16 mice model, and these drugs significantly enhanced anti-PD-1/anti-CTLA-4 therapy and prolonged survival [56].

Several drug combinations, such as PD-1/PD-L1 antibodies in combination with drugs targeting VEGF or TGF-β, have been approved for clinical trials in a variety of indications and achieved superior anticancer effects to anti-PD-1/PD-L1 monotherapy [57–59]. According to our in vivo findings, simultaneous blockade of PD-1, VEGF, and TGF-β exhibited significantly greater anticancer efficacy than either PD-1 plus VEGF or PD-1 plus TGF-β blockage. Bispecific antibodies are regarded as the next generation of immunotherapy strategies with broader anti-tumor spectrum and better therapeutic efficiency [60, 61]. In general, TGF-β and VEGF are abundantly expressed in tumor tissues [62]. The Nano-BODY™ platform-based development of bispecific antibody Y332D not only blocks both TGF-β and VEGF signaling pathways, but also establishes a physical link between the anti-TGF-β and anti-VEGF termini due to its unique molecular structure, allowing for better aggregation at the tumor site and exerting synergistic effects.

## Conclusions

In this study, we developed Y332D, the first novel bispecific antibody to block both TGF-β and VEGF signalings. Y332D demonstrated superior antitumor effectiveness to anti-TGF- β or anti-VEGF therapy alone in mouse tumor models. In addition, we had also developed a novel combination therapy regarding Y332D and anti-PD-1 antibody that stimulated antitumor immunity and exhibited superior anticancer efficacy to anti-PD-1 plus anti-VEGF or anti-PD-1 plus anti-TGF-β. Further investigations revealed that Y332D promoted the transformation of non-inflammatory into immunoinflammatory tumors and enhanced the responsiveness of anti-PD-1 antibody. These findings imply that this combination therapy strategy has a broad anticancer spectrum and may overcome resistance to anti-PD-1 therapy.

### Supplementary Information

**Additional file 1: Fig. S1.** Y332D antagonized TGF-β/Smad signaling and TGF-β-regulated epithelial-mesenchymal transition (EMT) in cancer cells. **a**, **b** SBE4 luciferase reporter assay was performed to test the blocking capability of Y332D on TGF-β/Smad signaling pathway. **c,** **d** Western blotting assay was performed to measure the antagonistic effect of Y332D on TGF-β-regulated EMT in cancer cells. *p   < 0.05, ***p < 0.001, and ****p < 0.0001 denote the significant difference relative to Y332D treatment. α-TGF-β: anti-TGF-β, α-VEGF: anti-VEGF. **Fig. S2.** Y332D inhibited lung metastasis in 4T1 murine tumor model. 2×10^4^ 4T1 cells were inoculated in the right mammary fat pad of BALB/c mice on day 0. Treatment started on day 14. Mice were euthanized and lung tissues were collected after inoculation for 34 days. **a**, **b** The number of 4T1 tumor nodules in lung tissues and representative images of H&E staining of lung tissues were shown. Bars, SDs; **p < 0.01 and ***p < 0.001 denote the significant difference relative to Y332D therapy. α-TGF-β: anti-TGF-β, α-VEGF: anti-VEGF. **Fig. S3.** Combination treatment is biologically safe in vivo. Tumor-bearing mice were treated for 11 days and euthanatized when the study ended. **a**–**c** Body weight of mice was measured every other day or every two days. **Fig. S4.** Flow cytometry assay to analyze tumor-infiltrating lymphocytes in H22 tumor model. The representative images and quantitative analysis of tumor-infiltrating **a** lymphocytes, **b** T cells, **c** Ki67^+^ T cells, **d** CD69^+^ T cells, **e** CD107a^+^ T cells, **f** Granzyme B^+^ T cells. The proportion of tumor-infiltrating immune cells in the total live cells was calculated. Bars, SDs; *p < 0.05, **p < 0.01, and ***p < 0.001 denote the significant difference relative to combination treatment. α-PD-1: anti-PD-1. **Fig. S5.** RNA-seq assay to explore the immune profile of H22 tumors after different treatments. **a** Significantly enriched immune-related Kyoto Encyclopedia of Genes and Genomes (KEGG) terms (α-PD-1+Y332D vs. Vehicle; α-PD-1+Y332D vs. α-PD-1; α-PD-1+Y332D vs. Y332D). **b**–**f** Gene set enrichment analysis (GSEA) plot (α-PD-1+Y332D vs. α-PD-1). group4: α-PD-1+Y332D, group2: α-PD-1. α-PD-1: anti-PD-1. NES: Normalized Enrichment Score.

## Data Availability

All data needed to evaluate the conclusions in the article are present in the article and/or the Supplementary Materials. The data and materials used in the current study are available from the corresponding authors upon reasonable request.

## Abbreviations

PD-1: Programmed cell death protein 1
PD-L1: Programmed death-ligand 1
TCR: T cell receptor
DC: Dendritic cell
MDSC: Myeloid-derived suppressor cell
TAM: Tumor-associated macrophages
CAF: Cancer-associated fibroblasts
HUVEC: Human umbilical vein endothelial cells
SDS-PAGE: Sodium dodecyl-sulfate polyacrylamide gel electrophoresis
CE-SDS: Capillary electrophoresis with sodium dodecylsulfate
SPR: Surface plasmon resonance
ELISA: Enzyme-linked immunosorbent assay
IF: Immunofluorescent
TILs: Tumor-infiltrating lymphocytes
EMT: Epithelial-mesenchymal transition

## References

[1] Boussiotis VA (2016). Molecular and biochemical aspects of the PD-1 checkpoint pathway. N Engl J Med.

[2] Liu Z, Yu X, Xu L, Li Y, Zeng C (2022). Current insight into the regulation of PD-L1 in cancer. Exp Hematol Oncol.

[3] Yang X, Ma L, Zhang X, Huang L, Wei J (2022). Targeting PD-1/PD-L1 pathway in myelodysplastic syndromes and acute myeloid leukemia. Exp Hematol Oncol.

[4] Freeman GJ, Long AJ, Iwai Y, Bourque K, Chernova T, Nishimura H (2000). Engagement of the PD-1 immunoinhibitory receptor by a novel B7 family member leads to negative regulation of lymphocyte activation. J Exp Med.

[5] Niu M, Liu Y, Yi M, Jiao D, Wu K (2022). Biological characteristics and clinical significance of soluble PD-1/PD-L1 and exosomal PD-L1 in cancer. Front Immunol.

[6] Sun C, Mezzadra R, Schumacher TN (2018). Regulation and function of the PD-L1 checkpoint. Immunity.

[7] Niu M, Yi M, Li N, Luo S, Wu K (2021). Predictive biomarkers of anti-PD-1/PD-L1 therapy in NSCLC. Exp Hematol Oncol.

[8] Jia Q, Wang A, Yuan Y, Zhu B, Long H (2022). Heterogeneity of the tumor immune microenvironment and its clinical relevance. Exp Hematol Oncol.

[9] Chen DS, Mellman I (2013). Oncology meets immunology: the cancer-immunity cycle. Immunity.

[10] Zhao H, Wu L, Yan G, Chen Y, Zhou M, Wu Y (2021). Inflammation and tumor progression: signaling pathways and targeted intervention. Signal Transduct Target Ther.

[11] Qin S, Yi M, Jiao D, Li A, Wu K (2020). Distinct roles of VEGFA and ANGPT2 in lung adenocarcinoma and squamous cell carcinoma. J Cancer.

[12] Ghalehbandi S, Yuzugulen J, Pranjol MZI, Pourgholami MH (2023). The role of VEGF in cancer-induced angiogenesis and research progress of drugs targeting VEGF. Eur J Pharmacol.

[13] Apte RS, Chen DS, Ferrara N (2019). VEGF in signaling and disease: beyond discovery and development. Cell.

[14] Jain RK (2003). Molecular regulation of vessel maturation. Nat Med.

[15] Böckelmann LC, Schumacher U (2019). Targeting tumor interstitial fluid pressure: Will it yield novel successful therapies for solid tumors?. Expert Opin Ther Targets.

[16] Kudo M (2020). Scientific rationale for combined immunotherapy with PD-1/PD-L1 antibodies and VEGF inhibitors in advanced hepatocellular carcinoma. Cancers.

[17] Fukumura D, Kloepper J, Amoozgar Z, Duda DG, Jain RK (2018). Enhancing cancer immunotherapy using antiangiogenics: opportunities and challenges. Nat Rev Clin Oncol.

[18] Wallin JJ, Bendell JC, Funke R, Sznol M, Korski K, Jones S (2016). Atezolizumab in combination with bevacizumab enhances antigen-specific T-cell migration in metastatic renal cell carcinoma. Nat Commun.

[19] Gabrilovich DI, Chen HL, Girgis KR, Cunningham HT, Meny GM, Nadaf S (1996). Production of vascular endothelial growth factor by human tumors inhibits the functional maturation of dendritic cells. Nat Med.

[20] Rahma OE, Hodi FS (2019). The intersection between tumor angiogenesis and immune suppression. Clin Cancer Res.

[21] Tie Y, Tang F, Wei Y, Wei X (2022). Immunosuppressive cells in cancer: mechanisms and potential therapeutic targets. J Hematol Oncol.

[22] Liu Z, Chen H, Zheng L, Sun L, Shi L (2023). Angiogenic signaling pathways and anti-angiogenic therapy for cancer. Signal Transduct Target Ther.

[23] Patel SA, Nilsson MB, Le X, Cascone T, Jain RK, Heymach JV (2023). Molecular mechanisms and future implications of VEGF/VEGFR in cancer therapy. Clin Cancer Res.

[24] Niu M, Yi M, Li N, Wu K, Wu K (2021). Advances of targeted therapy for hepatocellular carcinoma. Front Oncol.

[25] Liu J, Liu Q, Li Y, Li Q, Su F, Yao H (2020). Efficacy and safety of camrelizumab combined with apatinib in advanced triple-negative breast cancer: an open-label phase II trial. J Immunother Cancer.

[26] Lan C, Shen J, Wang Y, Li J, Liu Z, He M (2020). Camrelizumab plus apatinib in patients with advanced cervical cancer (CLAP): a multicenter, open-label, single-arm, phase II trial. J Clin Oncol.

[27] Fan Y, Zhao J, Wang Q, Huang D, Li X, Chen J (2021). Camrelizumab plus apatinib in extensive-stage SCLC (PASSION): a multicenter, two-stage, phase 2 trial. J Thorac Oncol.

[28] Liu X, Zhu X, Feng L, Li X, Xu B, Li K (2022). Physical activity improves outcomes of combined lenvatinib plus anti-PD-1 therapy in unresectable hepatocellular carcinoma: a retrospective study and mouse model. Exp Hematol Oncol.

[29] Bai X, Yi M, Jiao Y, Chu Q, Wu K (2019). Blocking TGF-β signaling to enhance the efficacy of immune checkpoint inhibitor. Onco Targets Ther.

[30] Chandra Jena B, Sarkar S, Rout L, Mandal M (2021). The transformation of cancer-associated fibroblasts: current perspectives on the role of TGF-β in CAF mediated tumor progression and therapeutic resistance. Cancer Lett.

[31] Yi M, Li T, Niu M, Wu Y, Zhao Z, Wu K (2022). TGF-β: a novel predictor and target for anti-PD-1/PD-L1 therapy. Front Immunol.

[32] Chen J, Gingold JA, Su X (2019). Immunomodulatory TGF-β signaling in hepatocellular carcinoma. Trends Mol Med.

[33] Batlle E, Massagué J (2019). Transforming growth factor-β signaling in immunity and cancer. Immunity.

[34] Yi M, Niu M, Zhang J, Li S, Zhu S, Yan Y (2021). Combine and conquer: manganese synergizing anti-TGF-β/PD-L1 bispecific antibody YM101 to overcome immunotherapy resistance in non-inflamed cancers. J Hematol Oncol.

[35] Yi M, Niu M, Wu Y, Ge H, Jiao D, Zhu S (2022). Combination of oral STING agonist MSA-2 and anti-TGF-β/PD-L1 bispecific antibody YM101: a novel immune cocktail therapy for non-inflamed tumors. J Hematol Oncol.

[36] Kim B-G, Malek E, Choi SH, Ignatz-Hoover JJ, Driscoll JJ (2021). Novel therapies emerging in oncology to target the TGF-β pathway. J Hematol Oncol.

[37] Mariathasan S, Turley SJ, Nickles D, Castiglioni A, Yuen K, Wang Y (2018). TGF-β attenuates tumour response to PD-L1 blockade by contributing to exclusion of T cells. Nature.

[38] Terabe M, Robertson FC, Clark K, De Ravin E, Bloom A, Venzon DJ (2017). Blockade of only TGF-β 1 and 2 is sufficient to enhance the efficacy of vaccine and PD-1 checkpoint blockade immunotherapy. Oncoimmunology.

[39] Xu L, Zou C, Zhang S, Chu TSM, Zhang Y, Chen W (2022). Reshaping the systemic tumor immune environment (STIE) and tumor immune microenvironment (TIME) to enhance immunotherapy efficacy in solid tumors. J Hematol Oncol.

[40] Ziani L, Buart S, Chouaib S, Thiery J (2021). Hypoxia increases melanoma-associated fibroblasts immunosuppressive potential and inhibitory effect on T cell-mediated cytotoxicity. Oncoimmunology.

[41] Yi M, Zhang J, Li A, Niu M, Yan Y, Jiao Y (2021). The construction, expression, and enhanced anti-tumor activity of YM101: a bispecific antibody simultaneously targeting TGF-β and PD-L1. J Hematol Oncol.

[42] Carlson CM, Frandsen JL, Kirchhof N, McIvor RS, Largaespada DA (2005). Somatic integration of an oncogene-harboring sleeping beauty transposon models liver tumor development in the mouse. Proc Natl Acad Sci USA.

[43] Lan Y, Zhang D, Xu C, Hance KW, Marelli B, Qi J (2018). Enhanced preclinical antitumor activity of M7824, a bifunctional fusion protein simultaneously targeting PD-L1 and TGF-β. Sci Transl Med.

[44] Bustin SA, Benes V, Garson JA, Hellemans J, Huggett J, Kubista M (2009). The MIQE guidelines: minimum information for publication of quantitative real-time PCR experiments. Clin Chem.

[45] Pfaffl MW (2001). A new mathematical model for relative quantification in real-time RT-PCR. Nucleic Acids Res.

[46] Liang W-C, Wu X, Peale FV, Lee CV, Meng YG, Gutierrez J (2006). Cross-species vascular endothelial growth factor (VEGF)-blocking antibodies completely inhibit the growth of human tumor xenografts and measure the contribution of stromal VEGF. J Biol Chem.

[47] Chou YT, Wang H, Chen Y, Danielpour D, Yang YC (2006). Cited2 modulates TGF-beta-mediated upregulation of MMP9. Oncogene.

[48] Hamerlik P, Lathia JD, Rasmussen R, Wu Q, Bartkova J, Lee M (2012). Autocrine VEGF-VEGFR2-Neuropilin-1 signaling promotes glioma stem-like cell viability and tumor growth. J Exp Med.

[49] Herbert SP, Stainier DYR (2011). Molecular control of endothelial cell behaviour during blood vessel morphogenesis. Nat Rev Mol Cell Biol.

[50] Heinolainen K, Karaman S, D'Amico G, Tammela T, Sormunen R, Eklund L (2017). VEGFR3 modulates vascular permeability by controlling VEGF/VEGFR2 signaling. Circ Res.

[51] Yi M, Wu Y, Niu M, Zhu S, Zhang J, Yan Y (2022). Anti-TGF-β/PD-L1 bispecific antibody promotes T cell infiltration and exhibits enhanced antitumor activity in triple-negative breast cancer. J Immunother Cancer.

[52] Nagarsheth N, Wicha MS, Zou W (2017). Chemokines in the cancer microenvironment and their relevance in cancer immunotherapy. Nat Rev Immunol.

[53] Cao Y, Jiao N, Sun T, Ma Y, Zhang X, Chen H (2021). CXCL11 correlates with antitumor immunity and an improved prognosis in colon cancer. Front Cell Dev Biol.

[54] Zhang J, Huang D, Saw PE, Song E (2022). Turning cold tumors hot: from molecular mechanisms to clinical applications. Trends Immunol.

[55] Galon J, Bruni D (2019). Approaches to treat immune hot, altered and cold tumours with combination immunotherapies. Nat Rev Drug Discov.

[56] Courau T, Nehar-Belaid D, Florez L, Levacher B, Vazquez T, Brimaud F (2016). TGF-β and VEGF cooperatively control the immunotolerant tumor environment and the efficacy of cancer immunotherapies. JCI Insight.

[57] Yi M, Zheng X, Niu M, Zhu S, Ge H, Wu K (2022). Combination strategies with PD-1/PD-L1 blockade: current advances and future directions. Mol Cancer.

[58] Yi M, Jiao D, Qin S, Chu Q, Wu K, Li A (2019). Synergistic effect of immune checkpoint blockade and anti-angiogenesis in cancer treatment. Mol Cancer.

[59] Yuan X, Yi M, Zhang W, Xu L, Chu Q, Luo S (2021). The biology of combination immunotherapy in recurrent metastatic head and neck cancer. Int J Biochem Cell Biol.

[60] Wu Y, Yi M, Zhu S, Wang H, Wu K (2021). Recent advances and challenges of bispecific antibodies in solid tumors. Exp Hematol Oncol.

[61] Yu S, Zhang J, Yan Y, Yao X, Fang L, Xiong H (2019). A novel asymmetrical anti-HER2/CD3 bispecific antibody exhibits potent cytotoxicity for HER2-positive tumor cells. J Exp Clin Cancer Res.

[62] Bu MT, Chandrasekhar P, Ding L, Hugo W (2022). The roles of TGF-β and VEGF pathways in the suppression of antitumor immunity in melanoma and other solid tumors. Pharmacol Ther.

