# Supplementary material for: Synergistic efficacy of simultaneous anti-TGF-β/VEGF bispecific antibody and PD-1 blockade in cancer therapy
Source: J Hematol Oncol. 2023 Aug 12;16:94. doi: 10.1186/s13045-023-01487-5 (PMC10423429; doi:10.1186/s13045-023-01487-5)
Supplement: Supplementary file 1 — Additional file 1: Fig. S1. Y332D antagonized TGF-β/Smad signaling and TGF-β-regulated epithelial-mesenchymal transition (EMT) in cancer cells. a, b SBE4 luciferase reporter assay was performed to test the blocking capability of Y332D on TGF-β/Smad signaling pathway. c, d Western blotting assay was performed to measure the antagonistic effect of Y332D on TGF-β-regulated EMT in cancer cells. *p < 0.05, ***p < 0.001, and ****p < 0.0001 denote the significant difference relative to Y332D treatment. α-TGF-β: anti-TGF-β, α-VEGF: anti-VEGF. Fig. S2. Y332D inhibited lung metastasis in 4T1 murine tumor model. 2×10^4^ 4T1 cells were inoculated in the right mammary fat pad of BALB/c mice on day 0. Treatment started on day 14. Mice were euthanized and lung tissues were collected after inoculation for 34 days. a, b The number of 4T1 tumor nodules in lung tissues and representative images of H&E staining of lung tissues were shown. Bars, SDs; **p < 0.01 and ***p < 0.001 denote the significant difference relative to Y332D therapy. α-TGF-β: anti-TGF-β, α-VEGF: anti-VEGF. Fig. S3. Combination treatment is biologically safe in vivo. Tumor-bearing mice were treated for 11 days and euthanatized when the study ended. a–c Body weight of mice was measured every other day or every two days. Fig. S4. Flow cytometry assay to analyze tumor-infiltrating lymphocytes in H22 tumor model. The representative images and quantitative analysis of tumor-infiltrating a lymphocytes, b T cells, c Ki67+ T cells, d CD69+ T cells, e CD107a+ T cells, f Granzyme B+ T cells. The proportion of tumor-infiltrating immune cells in the total live cells was calculated. Bars, SDs; *p < 0.05, **p < 0.01, and ***p < 0.001 denote the significant difference relative to combination treatment. α-PD-1: anti-PD-1. Fig. S5. RNA-seq assay to explore the immune profile of H22 tumors after different treatments. a Significantly enriched immune-related Kyoto Encyclopedia of Genes and Genomes (KEGG) terms (α-PD-1+Y332D vs. Vehicle; α [file 13045_2023_1487_MOESM1_ESM.docx]

**Synergistic efficacy of simultaneous anti-TGF-β/VEGF bispecific antibody and PD-1 blockade in cancer therapy**

**Mengke Niu^1^, Ming Yi^1,2^, Yuze Wu^1^, Lijuan Lyu^3^, Qing He^4^, Rui Yang^4^, Liang Zeng^4^, Jian Shi^4^, Jing Zhang^4^, Pengfei Zhou^4^, Tingting Zhang^5^, Qi Mei^1,5^*, Qian Chu^1^*, Kongming Wu^5,6^***

1. Department of Oncology, Tongji Hospital of Tongji Medical College, Huazhong University of Science and Technology, Wuhan, 430030, China.
2. Department of Breast Surgery, The First Affiliated Hospital, College of Medicine, Zhejiang University, Hangzhou, 310000, China.
3. Department of Oncology, The Second Affiliated Hospital of Xi’an Jiaotong University, Xi’an, 710000, China.
4. Wuhan YZY Biopharma Co., Ltd, Biolake, C2-1, No.666 Gaoxin Road, Wuhan, 430075, People's Republic of China.
5. Cancer Center, Shanxi Bethune Hospital, Shanxi Academy of Medical Science, Tongji Shanxi Hospital, Third Hospital of Shanxi Medical University, Taiyuan, 030032, China.
6. Cancer Center, Tongji Hospital of Tongji Medical College, Huazhong University of Science and Technology, Wuhan, 430030, China.

**Corresponding authors**

Kongming Wu, Cancer Center, Shanxi Bethune Hospital, Shanxi Academy of Medical Science, Tongji Shanxi Hospital, Third Hospital of Shanxi Medical University, Taiyuan, 030032, China. E-mail: [kmwu@tjh.tjmu.edu.cn](mailto:kmwu@tjh.tjmu.edu.cn).

Qian Chu, Department of Oncology, Tongji Hospital, Tongji Medical College, Huazhong University of Science and Technology, Wuhan, 430030, China. E-mail: qianchu@tjh.tjmu.edu.cn.

Qi Mei, Department of Oncology, Tongji Hospital, Tongji Medical College, Huazhong University of Science and Technology, Wuhan, 430030, China. E-mail: [borismq@163.com](mailto:borismq@163.com).

**Author’s e-mails:**

Mengke Niu: niumengke9505@163.com;

Ming Yi: [mingyi_onco@outlook.com](mailto:mingyi_onco@outlook.com);

Yuze Wu: wuyz0304@163.com;

Lijuan Lyu: [2805890069@qq.com](mailto:2805890069@qq.com);

Qing He: heqing@yzybio.com;

Rui Yang: yangrui@yzybio.com;

Liang Zeng: zengliang@yzybio.com;

Jian Shi: shijian@yzybio.com;

Jing Zhang: zhangjing@yzybio.com;

Pengfei Zhou: pfzhou@yzybio.com;

Tingting Zhang: 1102234585@qq.com;

Qi Mei: borismq@163.com;

Qian Chu: [qianchu@tjh.tjmu.edu.cn](mailto:qianchu@tjh.tjmu.edu.cn);

Kongming Wu: [kmwu@tjh.tjmu.edu.cn](mailto:kmwu@tjh.tjmu.edu.cn).


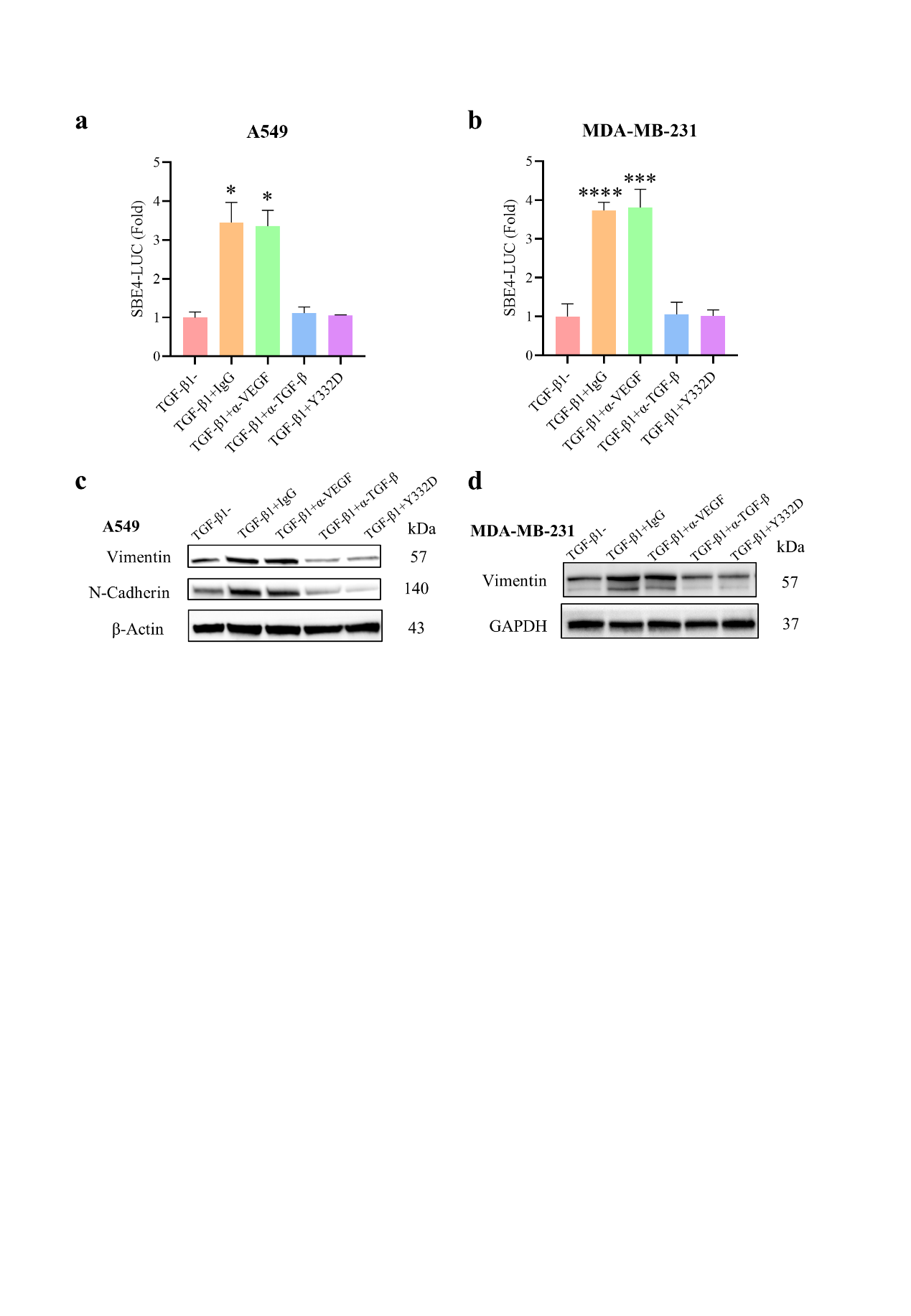


**Fig. S1 Y332D antagonized TGF-β/Smad signaling and TGF-β-regulated epithelial-mesenchymal transition (EMT) in cancer cells. a, b** SBE4 luciferase reporter assay was performed to test the blocking capability of Y332D on TGF-β/Smad signaling pathway. **c. d** Western blotting assay was performed to measure the antagonistic effect of Y332D on TGF-β-regulated EMT in cancer cells. **p* < 0.05, ***p* < 0.01, ****p* < 0.001, and *****p* < 0.0001 denote the significant difference relative to Y332D treatment. α-TGF-β: anti-TGF-β, α-VEGF: anti-VEGF


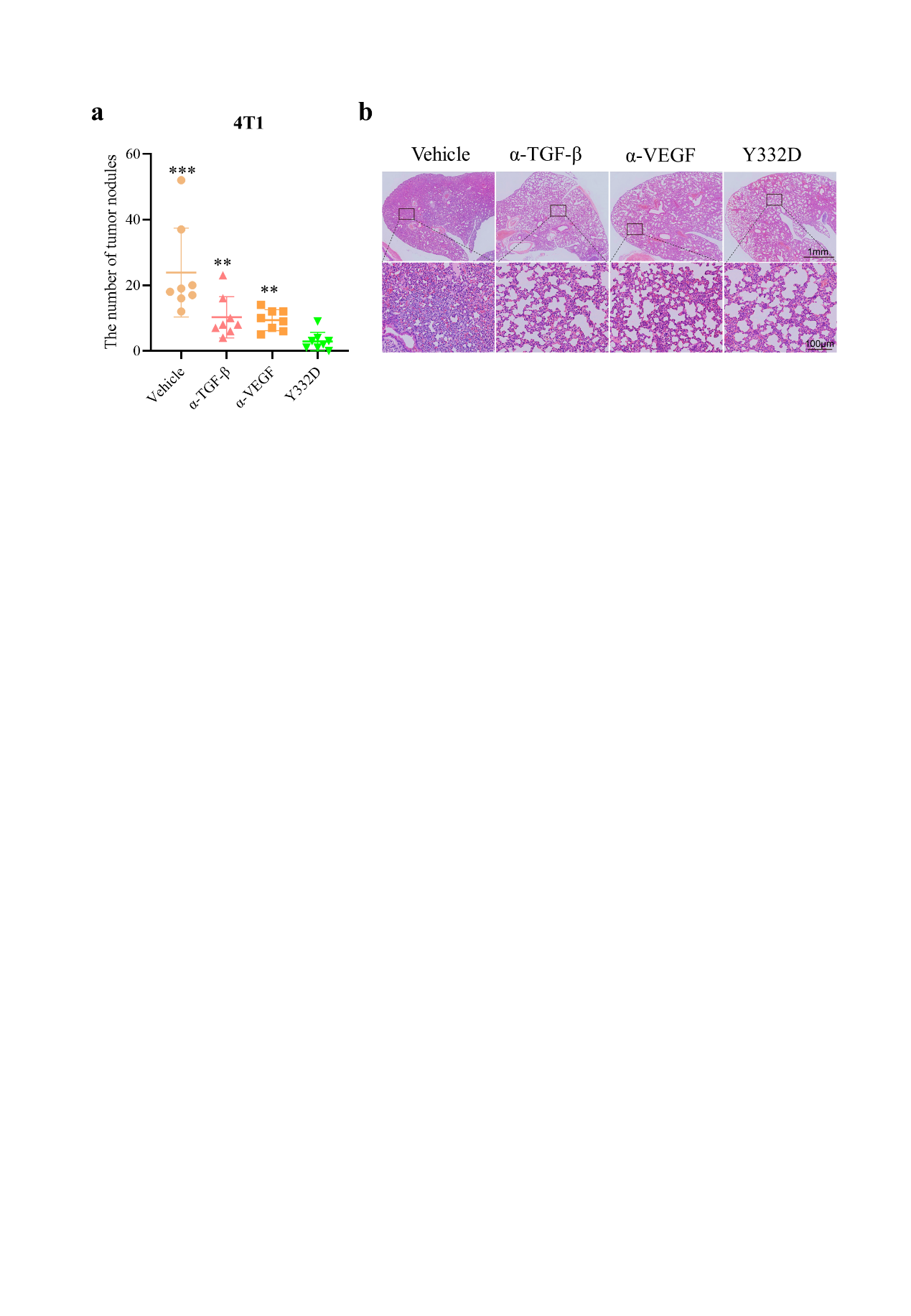


**Fig. S2 Y332D inhibited lung metastasis in 4T1 murine tumor model.** 2×10^4^ 4T1 cells were inoculated in the right mammary fat pad of BALB/c mice on day 0. Treatment started on day 14. Mice were euthanized and lung tissues were collected after inoculation for 34 days. **a, b** The number of 4T1 tumor nodules in lung tissues and representative images of H&E staining of lung tissues were shown. Bars, SDs; **p* < 0.05, ***p* < 0.01, ****p* < 0.001, and *****p* < 0.0001 denote the significant difference relative to Y332D therapy. α-TGF-β: anti-TGF-β, α-VEGF: anti-VEGF


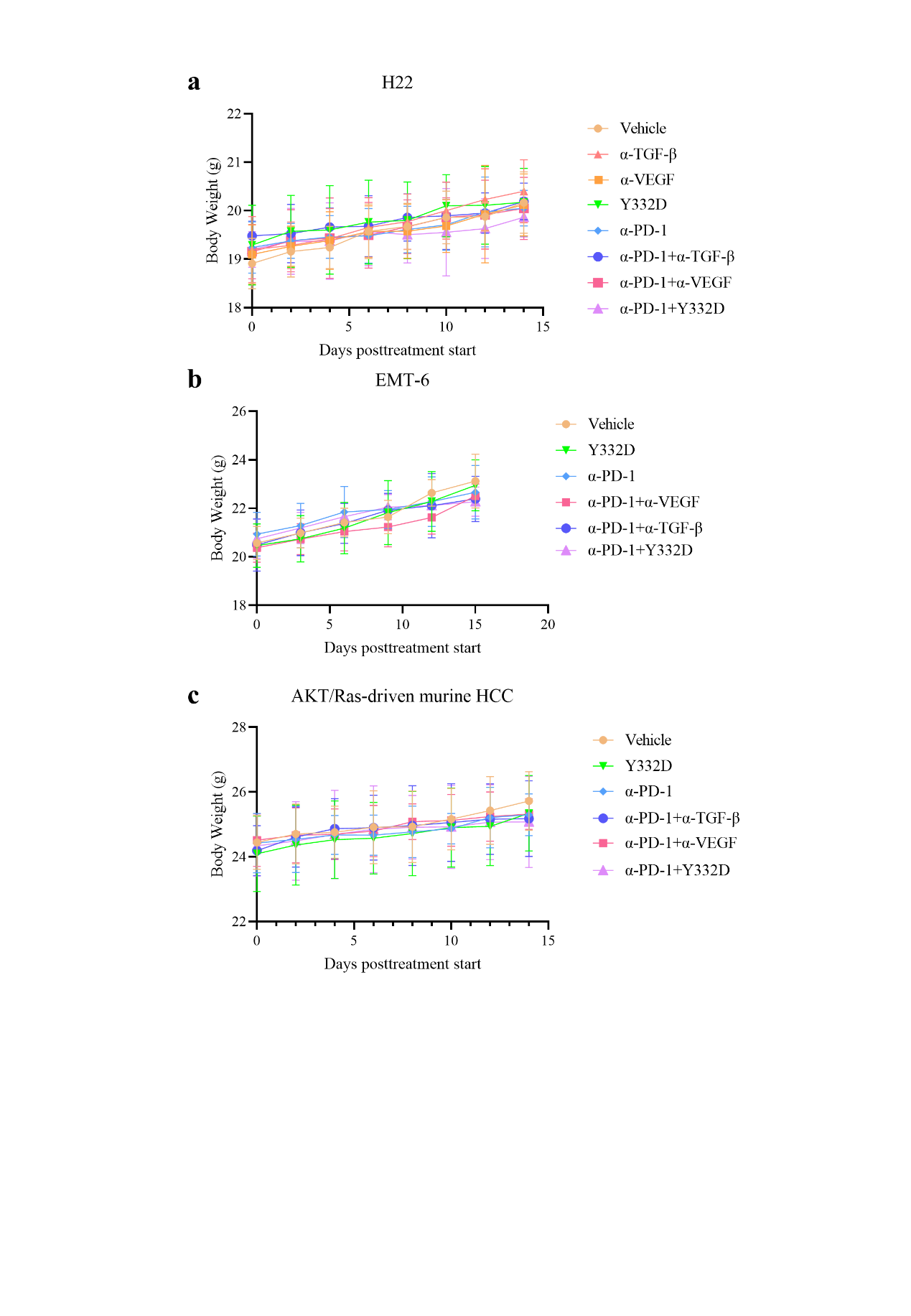


**Fig. S3 Combination treatment is biologically safe *in vivo*.** Tumor-bearing mice were treated for 11 days and euthanatized when the study ended. **a-c** Body weight of mice was measured every other day or every two days.


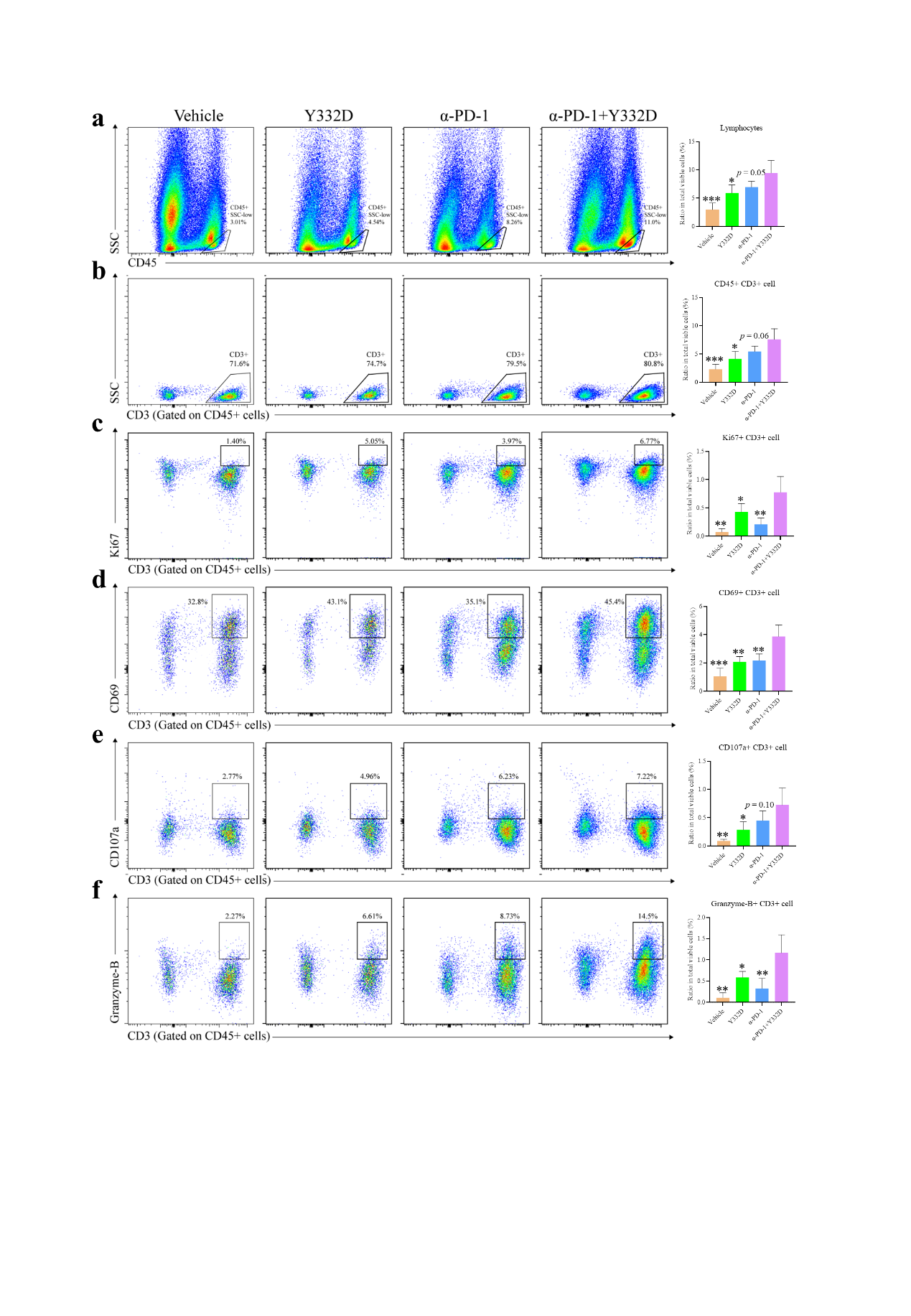


**Fig. S4 Flow cytometry assay to analyze tumor-infiltrating lymphocytes in H22 tumor model.** The representative images and quantitative analysis of tumor-infiltrating **a** lymphocytes, **b** T cells, **c** Ki67^+^ T cells, **d** CD69^+^ T cells, **e** CD107a^+^ T cells, **f** Granzyme B^+^ T cells. The proportion of tumor-infiltrating immune cells in the total live cells was calculated. Bars, SDs; **p* < 0.05, ***p* < 0.01, ****p* < 0.001, and *****p* < 0.0001 denote the significant difference relative to combination treatment. α-PD-1: anti-PD-1


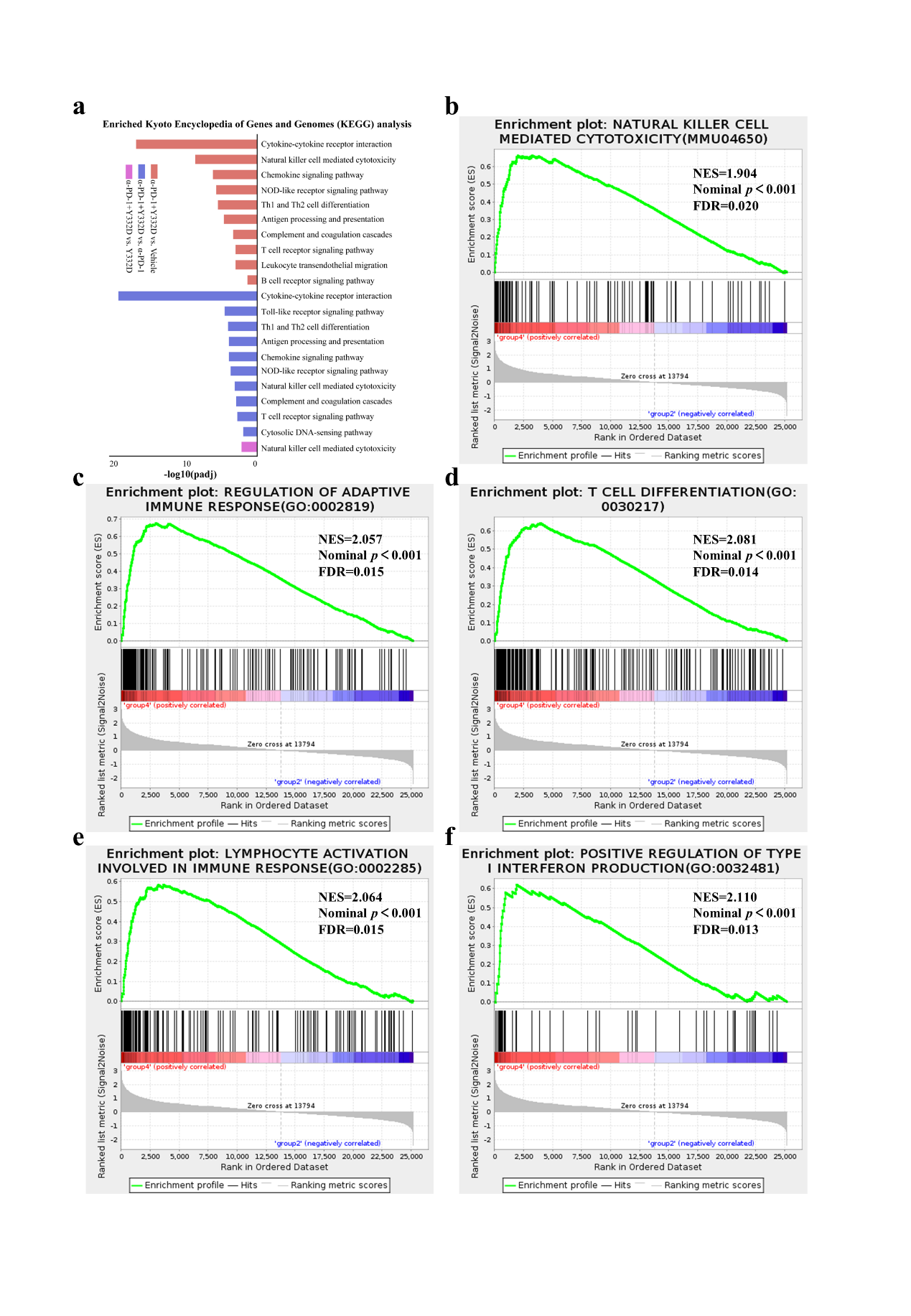


**Fig. S5: RNA-seq assay to explore the immune profile of H22 tumors after different treatments. a** Significantly enriched immune-related Kyoto Encyclopedia of Genes and Genomes (KEGG) terms (α-PD-1+Y332D vs. Vehicle; α-PD-1+Y332D vs. α-PD-1; α-PD-1+Y332D vs. Y332D). **b-f** Gene set enrichment analysis (GSEA) plot (α-PD-1+Y332D vs. α-PD-1). group4: α-PD-1+Y332D, group2: α-PD-1. α-PD-1: anti-PD-1. NES: Normalized Enrichment Score.
